# Two‐Dimensional Hydrazopyridinium Lead Iodide Perovskite Photodetectors With Broadband Sensitivity and Photoconductive Gain

**DOI:** 10.1002/smsc.70435

**Published:** 2026-10-28

**Authors:** Avija Ajayakumar, Akash Suresh, Andrzej Sławek, Anandhu Gopan, Chinnadurai Muthu, Amarjith V. Dev, Ashly Merlin Thomas, Anusree Aravindakshan, Konrad Szaciłowski, Chakkooth Vijayakumar

**Affiliations:** ^1^ Chemical Sciences and Technology Division CSIR‐National Institute for Interdisciplinary Science and Technology (NIIST) Thiruvananthapuram India; ^2^ Academy of Scientific and Innovative Research (AcSIR) Ghaziabad India; ^3^ Academic Centre for Materials and Nanotechnology AGH University of Krakow Krakow Poland; ^4^ Unconventional Computing Lab University of the West of England Bristol UK

**Keywords:** crystal engineering, Dion–Jacobson phase, low‐voltage operation, orthorhombic structure, trap‐mediated transport

## Abstract

Broadband photodetectors with enhanced ultraviolet sensitivity are critical for applications including environmental monitoring and optical communications, yet achieving high performance at low operating voltages remains challenging. Hydrazopyridinium lead iodide (HPDLI), a two‐dimensional Dion–Jacobson (DJ) perovskite synthesized via in situ reduction of 4,4′‐azopyridine, achieves a bandgap of 2.13 eV, among the lowest reported for *n = 1* lead halide systems, through an orthorhombic crystal structure with nearly linear Pb─I─Pb bonding (179.46°). This geometry maximizes metal‐halide orbital overlap, extending the broadband response toward the visible, with wavelength‐dependent absorption coefficients giving stronger sensitivity at shorter (deep‐UV) wavelengths. Lateral photodetectors exhibit 6 A/W responsivity, 1.01 × 10^12^ Jones shot‐noise‐limited detectivity, and 2900% external quantum efficiency at 254 nm under a low 0.2 V bias. High‐gain response is consistent with trap‐mediated photoconductive mechanisms and a proposed type‐II band alignment, where hydrazopyridinium cations may extend carrier lifetimes. Millisecond rise/fall times (40/70 ms), preliminary stability screening (2000‐s cyclic and continuous‐illumination protocols, a 48 h/80% RH switching‐shape comparison), and retention of characteristic PXRD peak positions after 5 months of ambient storage support crystal engineering as a route to energy‐efficient UV‐to‐visible photodetection, though longer, quantitative operational testing remains necessary.

## Graphical abstract

Hydrazopyridinium lead iodide combines near‐linear Pb─I─Pb bonding with a 2.13 eV bandgap in a two‐dimensional Dion–Jacobson perovskite. Spacer‐controlled crystal engineering enables broadband UV‐to‐visible photodetection with enhanced deep‐UV sensitivity. At just 0.2 V bias, lateral devices deliver 6 A W^−1^ responsivity and 2900% external quantum efficiency at 254 nm, demonstrating substantial photoconductive gain for low‐voltage optoelectronic sensing.

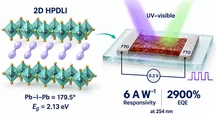

## 1 Introduction

Ultraviolet photodetection underpins critical technologies spanning environmental monitoring, secure optical communications, flame sensing, and biomedical diagnostics, driving demand for high‐performance semiconductor materials [1, 2]. Metal halide perovskites have emerged as candidates for photodetectors owing to their optoelectronic properties, including absorption coefficients approaching 10^4^ cm^−1^, carrier diffusion lengths exceeding 100 μm in single crystals, and defect‐tolerant electronic structures [3, 4, 5]. Solution processability enables low‐cost, large‐area device fabrication at temperatures below 150 °C, circumventing expensive vacuum‐based deposition required for conventional semiconductors [6]. Three‐dimensional (3D) perovskites such as MAPbI_3_, FAPbI_3_, and CsPbBr_3_ have demonstrated photodetector detectivities reaching 10^14^ Jones [3, 7].

3D perovskite photodetectors face challenges rooted in structural and chemical instability. Methylammonium lead iodide undergoes irreversible degradation upon exposure to moisture above 50% relative humidity, forming hydrated intermediates that decompose to PbI_2_ [8, 9]. Thermal instability initiates at temperatures as low as 80–100 °C, limiting operational conditions for device longevity [10]. Ion migration under an applied bias induces hysteresis, dark current drift, and gradual performance deterioration [11]. The accessible bandgap range in 3D lead halide perovskites (1.55 eV for MAPbI_3_ to 2.3 eV for MAPbBr_3_) constrains spectral tunability for UV‐enhanced detection [12]. To mitigate the intrinsic moisture and thermal instabilities of 3D perovskites, lower‐dimensional counterparts—particularly two‐dimensional (2D) Ruddlesden–Popper (RP) and Dion–Jacobson (DJ) layered metal halides—have drawn increasing attention. The natural quantum‐well architecture of 2D metal halides confers unique advantages, including suppressed ion migration, tunable exciton binding energies, and hydrophobic organic spacer layers that improve environmental stability [13, 14]. Spacer engineering extends beyond bandgap tuning: in noncentrosymmetric bromide perovskites, halogen substitution or a “molecular scissor” spacer redesign tunes ferroelectric polarization and photovoltaic response, illustrating the broader design space—though not applicable to the centrosymmetric iodide system reported here [15, 16]. However, single‐layer (*n = 1*) lead iodide systems typically exhibit wide bandgaps in the 2.3–2.6 eV range—for instance, (BA)_2_PbI_4_ at 2.43 eV and (PEA)_2_PbI_4_ at approximately 2.46 eV [17, 18]. Achieving lower bandgaps in *n = 1* systems while maintaining 2D structural stability and preferential UV response represents a key materials design challenge requiring innovative crystal engineering approaches.

Electronic properties of 2D‐layered perovskites are sensitive to inorganic layer geometry, which can be tuned through organic spacer cation selection [19, 20]. The Pb─I─Pb bond angle serves as the critical structural parameter governing bandgap: octahedral tilting deviations from the ideal 180° reduce orbital overlap between Pb 6s/6p and I 5p states, narrowing electronic bandwidth and widening the optical bandgap [21, 22]. Conventional alkylammonium spacers such as butylammonium and phenethylammonium induce substantial octahedral tilting, yielding in‐plane Pb─I─Pb angles of 150–155° that maintain bandgaps above 2.3 eV [18, 23]. Crystal symmetry influences carrier transport properties, with orthorhombic phases generally exhibiting more symmetric octahedral arrangements and enhanced orbital overlap compared to monoclinic phases prevalent in many 2D systems [24]. Conjugated aromatic spacers can template highly linear Pb─I─Pb geometries approaching 180° through optimized hydrogen bonding interactions, enabling systematic bandgap reduction in 2D lead iodide perovskites while reducing dielectric mismatch between organic and inorganic components [25, 26, 27].

Hydrazopyridinium lead iodide (HPDLI), an *n = 1* Dion–Jacobson perovskite synthesized via in situ reduction of 4,4′‐azopyridine in concentrated hydroiodic acid, exploits spacer‐controlled octahedral geometry to achieve a bandgap of 2.13 eV (absorption onset near 582 nm), among the lowest reported for *n = 1* 2D lead iodide systems. This is enabled by near‐linear Pb─I─Pb bond angles of 179.46°, with an octahedral angle variance (*σ* ^2^ = 40.48 deg^2^, quadratic elongation *λ* = 1.0117), reflecting enhanced σ‐ and π‐orbital overlap within the inorganic framework. This near‐linear bridging geometry, rather than orthorhombic symmetry in general, is what favors the enhanced orbital overlap and narrower bandgap observed here. The resulting narrow bandgap extends the response toward the visible; wavelength‐dependent absorption coefficients yield stronger sensitivity at shorter, deep‐UV wavelengths. Systematic characterization of structural, optical, and photoelectric properties demonstrates that spacer cation design extends the accessible bandgap range while enabling high‐gain, energy‐efficient photodetection.

## 2 Results and Discussion

HPDLI was synthesized via slow‐cooling crystallization by dissolving lead iodide (PbI_2_) and 4,4′‐azopyridine in a 1:2 molar ratio (PbI_2_:APD, APD in a twofold molar excess) in aqueous hydroiodic acid (57 wt%) at 130 °C, followed by gradual cooling at 5 °C/h to yield dark red, square sheet‐like crystals. The concentrated hydroiodic acid serves simultaneously as a solvent, protonating agent, and reducing medium: it dissolves the inorganic precursor while protonating the pyridine nitrogen atoms and reducing the azo linkage (─N═N─) of 4,4′‐azopyridine to a hydrazo linkage (─NH─NH─), generating a divalent hydrazopyridinium cation in situ. This divalent cation directs formation of the Dion–Jacobson (DJ) phase, in which one hydrazopyridinium dication charge‐balances one [PbI_4_]^2−^ layer, rather than the two monovalent cations per layer that define a Ruddlesden–Popper (RP) phase. Single‐crystal X‐ray diffraction confirmed this two‐dimensional layered architecture, with hydrazopyridinium moieties flanking the inorganic octahedral layers (Figure 1a).

**FIGURE 1:**
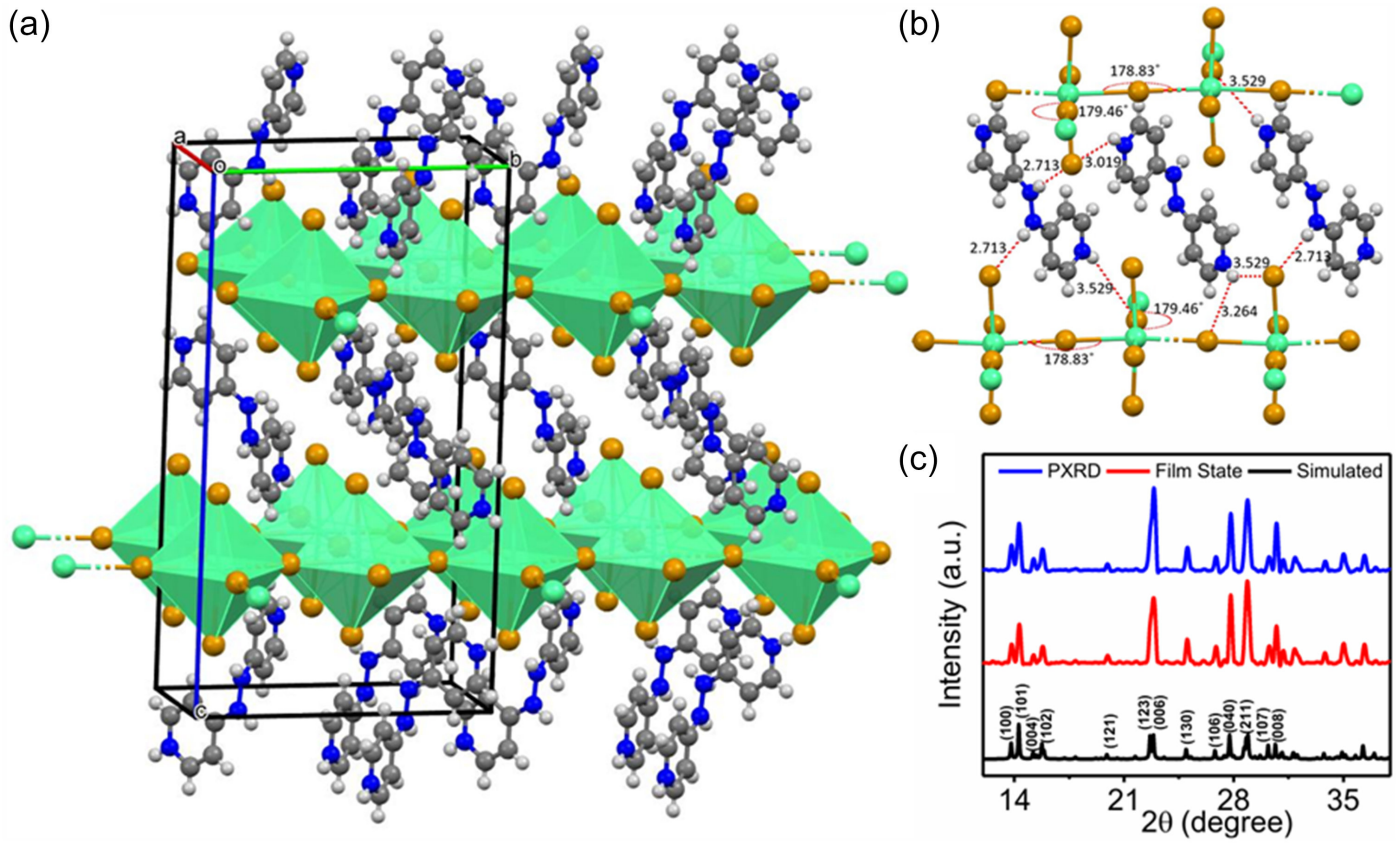
Crystal structure, synthesis, and phase purity of HPDLI. (a) Crystal structure and unit cell representation of HPDLI (Pb: cyan, I: dark yellow, N: blue, C: dark gray, H: light gray, PbI_6_^4−^ octahedra shown in cyan). (b) Bond angles and the H‐bonding interactions, with corresponding distances depicted in a ball‐and‐stick representation. (c) X‐ray diffraction (XRD) patterns of powder and thin‐film samples, alongside the simulated single‐crystal XRD pattern, with Miller indices assigned for the simulated diffraction peaks.

In situ reduction and protonation of 4,4′‐azopyridine to the divalent hydrazopyridinium cation (HPD) during single‐crystal growth in concentrated HI are supported by ^1^H‐NMR, FTIR, and Raman spectroscopy. In the ^1^H‐NMR spectrum (DMSO‐d_6_), while the 4,4′‐azopyridine precursor displays only sharp aromatic doublets at δ = 8.91 ppm (H_a_) and δ = 7.83 ppm (H_b_) (Figure S1), the dissolved HPDLI crystals exhibit two distinct downfield broad singlets at δ = 10.75 ppm (2H) and δ = 13.78 ppm (2H) (Figure S2). These resonances correspond to the hydrazo (─NH─NH─) protons and the protonated pyridinium (─NH^+^) nitrogens, respectively. Concurrently, the aromatic protons adjacent to the central linkage (H_b_) shift upfield to δ = 7.11 ppm owing to the electron‐donating effect of the reduced hydrazo spacer. FTIR spectra (Figure S3) further substantiate this conversion via the appearance of broad hydrazo/pyridinium ─NH─ stretching vibrations at 3150–3350 cm^−1^ and the loss of the characteristic azo N═N mode at 1415 cm^−1^. Raman spectra confirm the presence of hydrazo N─N stretching vibrations at ≈1250 cm^−1^ (Figure S4). Together with the refined single‐crystal N─N bond length of 1.379(7) Å—shorter than the N═N double bond of the 4,4′‐azopyridine precursor, consistent with reduction to a hydrazo (─NH─NH─) single bond; the N─H proton positions were geometrically constrained in the refinement rather than independently located—these results support the divalent hydrazopyridinium cation as the organic spacer templating the 2D Dion–Jacobson lattice.

The material crystallizes in an orthorhombic (Pbcm) space group with nearly linear Pb─I─Pb bridging angles of 179.46°, which govern the band‐edge orbital overlap, and exhibits extensive hydrogen‐bonding interactions within the crystal structure (Figure 1b). Quantitative structural analysis (Supporting Information) yields an octahedral angle variance of *σ* ^2^ = 40.48 deg^2^ and a quadratic elongation of *λ* = 1.0117—intraoctahedral distortion metrics, distinct from the interoctahedral bridging angle mentioned above. Phase purity and structural retention upon thin‐film processing were confirmed through powder and thin‐film X‐ray diffraction analysis, which showed agreement with the simulated single‐crystal diffraction pattern (Figure 1c). SEM analysis reveals a layered morphology in both crystal and film samples, consistent with the layered crystal structure established by single‐crystal X‐ray diffraction (Figure S5). Energy‐dispersive X‐ray spectroscopy (EDS) detects the expected constituent elements (Pb, I, N, C), with the measured atomic composition treated as semiquantitative given the known limitations of EDS for light elements (C, N) and for the heavy/light element pair I/Pb (Figure S6); elemental mapping images show a uniform spatial distribution of constituent elements within the imaged field of view (Figure S7). Refinement in this orthorhombic (Pbcm) setting achieves an *R*‐factor of 1.85% [*R*1, *I* > 2*σ*(*I*)], reflecting close agreement between observed and calculated structure factors, compared with the previously reported monoclinic description (P2/c, *R*1 = 10.19%; Table S1).

Optical characterization performed on powder samples revealed that HPDLI exhibits strong absorption across the visible spectrum to approximately 575 nm, with an absorption tail extending to ~620 nm (Figure 2a). Absorption coefficients increase progressively toward shorter wavelengths, with particularly strong absorption in the UV region. Tauc plot analysis of the F(R) data yielded a direct‐transition Tauc estimate of 2.13 eV, with a secondary onset at 2.07 eV of unresolved assignment; a sub‐bandgap or excitonic origin is plausible, consistent with the pronounced excitonic character of quantum‐confined 2D perovskites (Figure 2b) [28]. To understand this electronic property in the context of dimensional and structural engineering, a comparison of optical bandgaps and in‐plane Pb─I─Pb bond angles for reported *n = 1* 2D layered lead iodide perovskites is summarized in Figure 2d and compiled in Table S2. As demonstrated across these systems, the optical bandgap shows a broad inverse association with the in‐plane Pb─I─Pb bond angle (Pearson *r* ≈ −0.71, *R* ^2^ ≈ 0.50 across the 31 entries in Table S2 with both quantities reported), with substantial scatter arising from spacer chemistry, dielectric environment, and other lattice distortions; this is not treated as a strict one‐parameter law. As the bond angle approaches the ideal 180° limit, structural distortions are minimized, maximizing Pb(6s)‐I(5p) orbital overlap and resulting in bandgap narrowing. Within this comparison, the near‐linear Pb─I─Pb bond angle (179.46°) places HPDLI’s optical bandgap (2.13 eV) among the lowest reported for *n = 1* 2D lead iodide perovskites, comparable to the 2.19 eV bandgap reported for the closely related, near‐180° (APD)PbI_4_ system by Pariari et al. [27].

**FIGURE 2:**
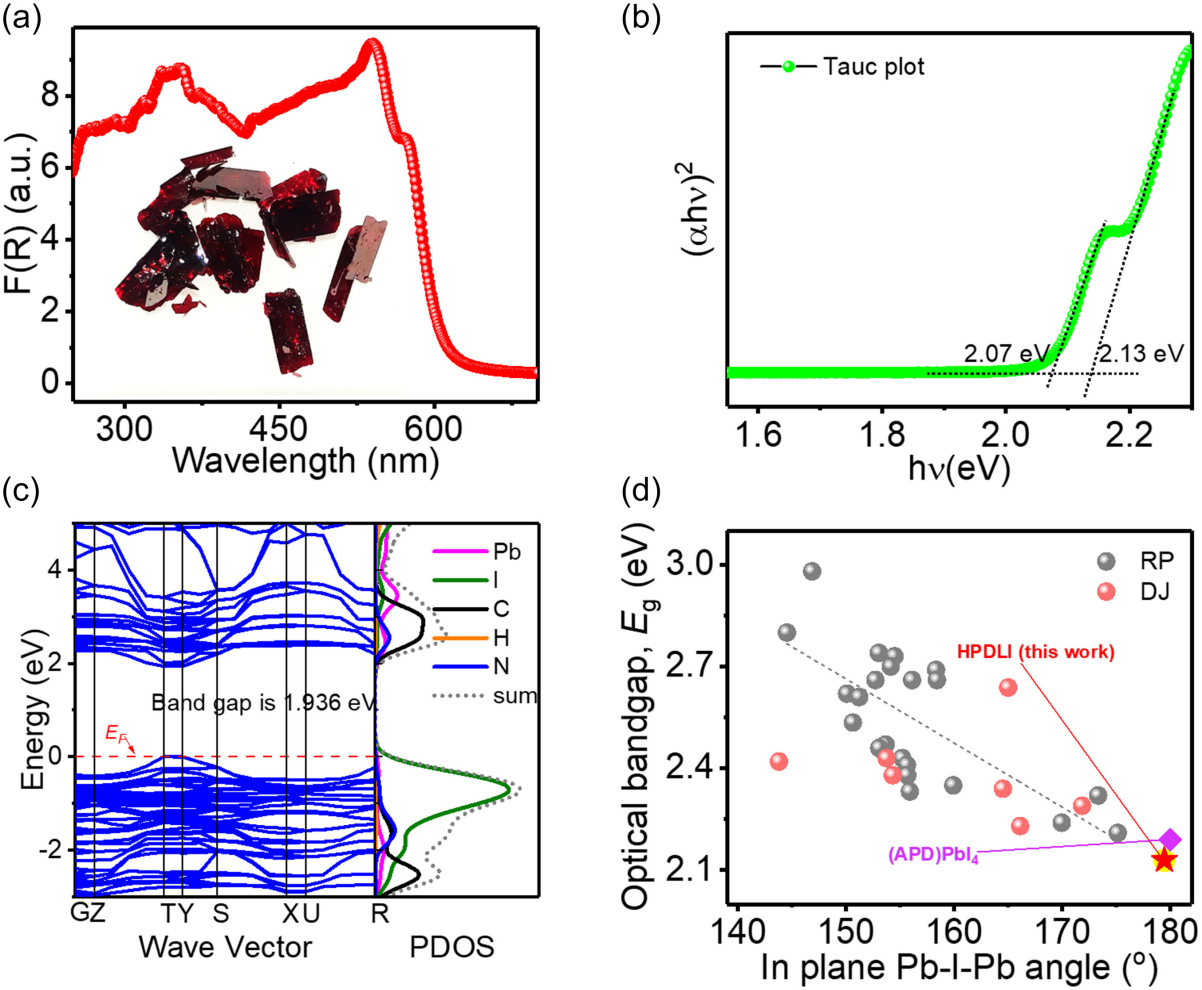
Optical and electronic band structure of HPDLI. (a) UV–vis diffuse‐reflectance spectrum of HPDLI, transformed into the Kubelka–Munk remission function F(R) (see Experimental Section, Supporting Information), with inset showing single crystals. (b) Tauc plot showing direct optical bandgap extraction (*E*_g_ = 2.13 eV) with an excitonic/sub‐bandgap onset at 2.07 eV. (c) Electronic structure analysis: total density of states (TDOS), partial density of states (PDOS), and calculated band structure. (d) Optical bandgap versus in‐plane Pb─I─Pb angle for reported *n = 1*, corner‐sharing 2D lead iodide perovskites (RP and DJ, distinguished by marker type); entries lacking a reported angle are omitted from this comparison. HPDLI (this work) and the related (APD)PbI_4_ report are highlighted. Bandgap values and primary references are listed in Table S2.

Density functional theory calculations yield a direct Kohn–Sham bandgap of 1.936 eV (Figure 2c), where the underestimation relative to the experimental value is consistent with well‐documented GGA functional behavior [29]. This computed value is used only to assess the qualitative orbital character of the band edges, not as a quantitative match to the experimental optical gap; full methodological details are provided in the Supporting Information. The calculated electronic structure projects a type‐II‐like band alignment between organic and inorganic sublattices, with contributions from hydrazopyridinium cation orbitals to the conduction band minimum, indicating that these moieties participate in the electronic structure rather than acting solely as structural spacers. This contribution is attributed to the electron‐acceptor character of pyridinium cations [30], and the resulting organic–inorganic electronic coupling is consistent with—though does not by itself establish—the trap‐mediated photoconductive gain discussed below, in which organic‐associated states would act as long‐lived traps for one carrier type, extending carrier lifetimes beyond the transit time.

To experimentally probe the electronic band positions predicted by DFT, ultraviolet photoelectron spectroscopy (UPS), low‐energy inverse photoemission spectroscopy (LEIPS), and X‐ray photoelectron spectroscopy (XPS) were performed (Figures S8–S11 and Table S3). Linear extrapolation of the UPS and LEIPS spectral onsets yields a valence band maximum (VBM) of −6.056 eV (ionization potential = 6.056 eV) and a conduction band minimum (CBM) of −2.794 eV (electron affinity = 2.794 eV) relative to the vacuum level, with a work function of *Φ* = 4.5 eV (Figures S9 and S10). The energy difference corresponds to a surface‐accessible electronic gap (*E*_g,el_) of 3.262 eV, reported as 3.26 eV; this value is obtained from the unrounded UPS/‍LEIPS onsets above and is not independently recalculated from rounded display values. UPS and LEIPS are inherently surface‐sensitive techniques, probing only the outermost few nanometers of the material, and do not resolve organic‐versus‐inorganic orbital character at the band edges; this value is accordingly treated as a surface‐accessible transport gap rather than a direct, bulk‐equivalent measure of the optical (*E*_g,opt_ = 2.13 eV) or DFT‐computed (1.936 eV) gaps reported above and is not used to infer an exciton binding energy from their difference. Nor does it, on its own, validate the type‐II organic/inorganic alignment proposed from DFT; that alignment (a qualitative prediction, as noted above) is retained as a hypothesis consistent with the available evidence rather than one confirmed by it, with transient optical or electrical spectroscopy identified as the appropriate further test. In comparison to the prepatterned FTO contacts (*Φ*_FTO_ = 4.3 eV, CBM ≈ −3.5 eV), a difference between the listed conduction‐band onsets of Δ*E*_C_ ≈ 0.71 eV is obtained (Figure S11 scheme); this onset difference is not itself a measured Schottky injection barrier (*Φ*_Bn_). This offset is consistent with suppressed dark‐carrier injection under the low operating bias used here (0.2 V) alongside efficient extraction of photogenerated carriers, although the present data do not independently establish a kinetically operative injection barrier or interfacial dipole.

Devices were fabricated using an FTO/HPDLI/FTO lateral architecture for direct assessment of intrinsic material properties without complications from additional charge‐transport layers that could introduce parasitic filtering effects. The devices were prepared by drop‐casting HPDLI thin films (500‐nm thickness) across a prepatterned lateral FTO channel defined by a channel length *L* = 10 µm and width *W* = 1 cm, yielding an effective active area *A* = *L* × *W* = 10^−3^ cm^2^. The photoconductive mode of operation yields broadband spectral response, as photons with energies exceeding the bandgap generate charge carriers regardless of wavelength [31, 32]. Wavelength‐dependent variation in photocurrent primarily reflects differences in the absorption coefficient and penetration depth rather than intrinsic spectral selectivity [33].

Figure 3a shows approximately symmetric, nonlinear |*I*|*–V* behavior in the dark and under UV illumination on a semi‐logarithmic scale; a corresponding signed, linear‐scale *I–V* plot confirming conduction symmetry and polarity across ±0.7 V is provided in Figure S12. Because the 254‐ and 365‐nm measurements were obtained at different irradiances, the separation between their raw current curves is not used as a quantitative measure of spectral sensitivity. Light illumination produced photocurrent enhancement, with 254‐nm UV light (*P*_in_ = 80 µW/cm^2^) generating higher currents than 365‐nm illumination (*P*_in_ = 140 µW/cm^2^) despite lower incident power, consistent with enhanced absorption at shorter wavelengths (see below for a wavelength‐resolved comparison at matched bias and irradiance). Systematic bias voltage optimization (Figure 3b) identified 0.2 V as the optimal operating point, where photocurrent maximizes while dark current remains minimal (109 nA), indicating efficient carrier extraction with minimal thermally generated background current. Beyond 0.3 V, both photocurrent and dark current increase substantially—with dark current rising superlinearly (reaching 320 nA at 0.5 V) and outpacing the light current, so that net photocurrent (*I*_ph_ = *I*_light_ − *I*_dark_) and therefore *R* and *D* ^*^_shot_ decline relative to their 0.2 V values—consistent with a growing contact/injection contribution at higher field; the present data support this picture but do not uniquely establish trap‐assisted tunneling as the specific injection mechanism.

**FIGURE 3:**
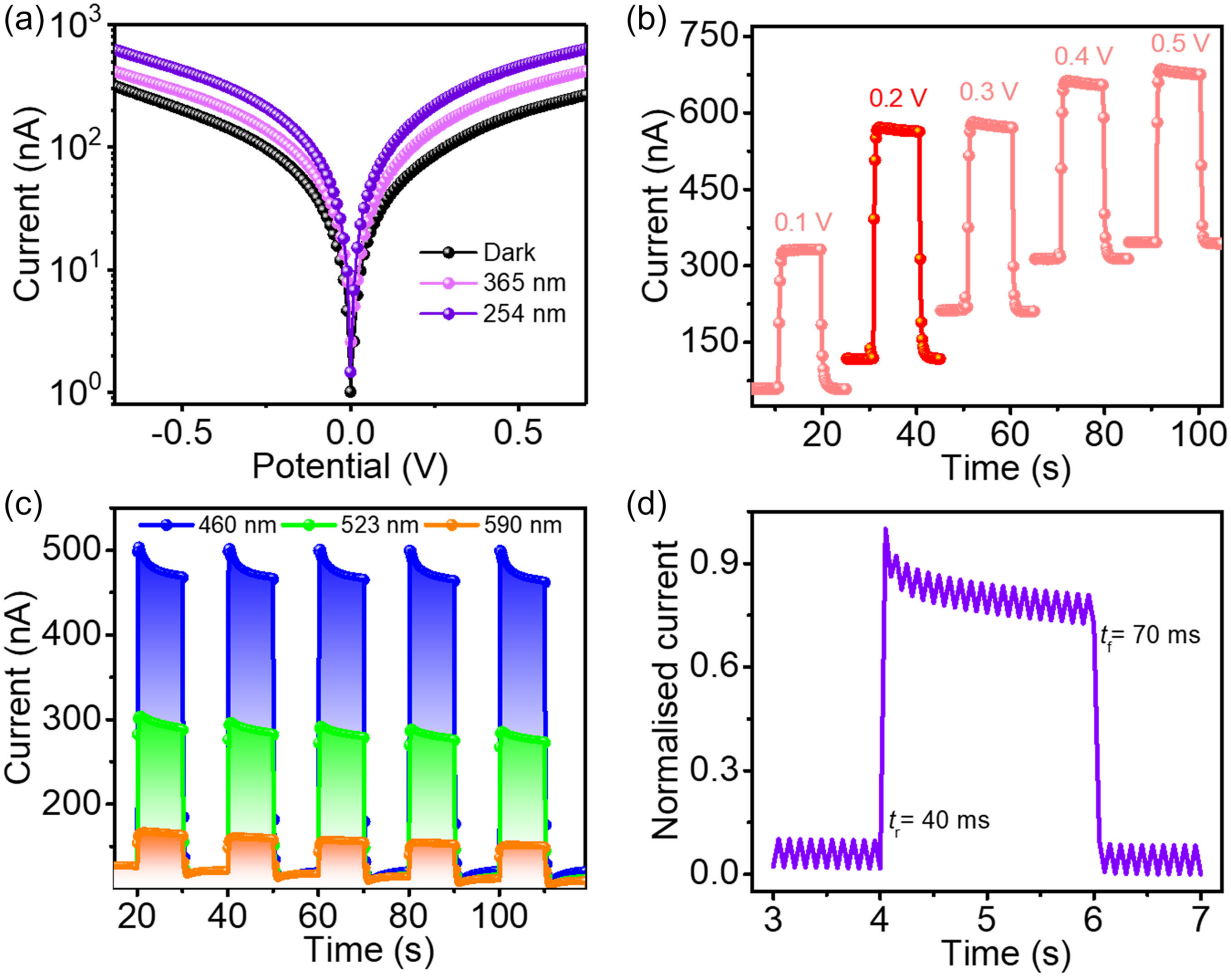
Photoresponse characteristics of HPDLI lateral photodetectors. (a) Absolute current–voltage (*I* *–V*) characteristics under dark conditions and UV illumination at 254 nm (*P*_in_ = 80 µW/cm^2^) and 365 nm (*P*_in_ = 140 µW/cm^2^). (b) Temporal photoresponse under 254‐nm illumination (*P*_in_ = 80 µW/cm^2^) at bias voltages from 0.1 V to 0.5 V. (c) Photoresponse under illumination across different wavelengths (460, 523, and 590 nm) at an applied bias of *V*_b_ = 0.2 V and an incident optical power density of *P*_in_ = 80 µW/cm^2^. (d) Dynamic temporal photoresponse recorded under 254‐nm illumination (*P*_in_ = 80 µW/cm^2^, *V*_b_ = 0.2 V) illustrating rise (40 ms) and fall (70 ms) times.

Wavelength‐dependent photoresponse measurements at a 0.2 V bias (Figure 3c) showed photocurrent decreasing systematically from 480 nA at 254 nm to 385 nA at 460 nm, 194 nA at 523 nm, and 45 nA at 590 nm. The broadband response was further verified through bias‐dependent *I–V* characterization under visible‐light excitation (460, 523, and 590 nm, each at matched *P*_in_ = 80 µW/cm^2^), which exhibited consistent photocurrent generation exceeding dark current levels across the visible spectrum (Figure S13). This wavelength‐dependent response differs from true UV‐selective photodetection. In solar‐blind or visible‐blind UV photodetectors, bandgap engineering provides intrinsic cutoff wavelengths that reject longer‐wavelength radiation, a characteristic determined by electronic structure rather than absorption coefficient variations [34, 35]. Commercial GaN‐based UV photodetectors achieve rejection ratios exceeding 10^4^–10^6^ through bandgap‐defined cutoff wavelengths [36], representing a fundamentally different detection mechanism. HPDLI, with a 2.13 eV bandgap (582‐nm cutoff), functions as a broadband photodetector: within the above‐bandgap range (254–523 nm), the wavelength‐dependent photocurrent reflects the wavelength dependence of absorption coefficients rather than intrinsic spectral selectivity. The response observed at 740 nm—below the nominal 2.13 eV/582 nm absorption edge—cannot be explained by this above‐bandgap mechanism and is reported here without a confirmed origin; possible contributions include sub‐bandgap/defect‐state absorption, measurement background, or instrumental leakage, none of which have been independently distinguished in this work.

The photodetector exhibited a rise and fall times of 40 and 70 ms, respectively (Figure 3d). As HPDLI crystallizes in the centrosymmetric space group Pbcm (point group mmm), a photo‐pyroelectric contribution to these transients is symmetry‐forbidden. The transient current spikes observed upon abrupt shuttering are consistent with photoinduced charge‐storage and polarization currents associated with charge restructuring across the electrode–film interface, together with carrier or ionic dynamics not isolated by this measurement, before the device settles into steady‐state conduction [37]. Minor signal fluctuations in the temporal response are attributed to transient trap‐filling and releasing processes characteristic of high‐gain photoconductors. These millisecond‐scale response times reflect the gain‐bandwidth tradeoff inherent to photoconductive devices, where extended carrier lifetimes that enable high gain simultaneously limit temporal response.

At the optimized 0.2 V bias, the photodetector achieved a responsivity of 6 A/W and a shot‐noise‐limited specific detectivity (*D* ^*^_shot_, an idealized estimate rather than a directly measured noise‐limited value; see the Supporting Information) of 1.01 × 10^12^ Jones (Figure 4). Statistical evaluation across five independently fabricated devices within one fabrication batch yields narrow error margins, confirming reproducible device‐to‐device performance, as plotted directly in Figure 4a (mean ± s.d., with *D* ^*^_shot_ computed for each device before averaging; error bars for responsivity are smaller than the data markers). Both parameters peaked at this voltage before declining due to increased dark current at higher biases (Figure 4a), indicating that carrier injection becomes dominant over photogeneration at elevated fields. Power‐dependent measurements (Figure S14) demonstrated a positive correlation between illumination intensity (25–80 µW/cm^2^) and device performance; because this ~3.2‐fold intensity range is comparatively narrow and active‐layer temperature was not measured, this is reported as a within‐range trend rather than evidence for a specific trap‐saturation mechanism or power‐law exponent (intensity‐dependent photocurrent is provided in Figure S15). This high‐gain response is consistent with a type‐II band alignment, where trapping of one carrier type in hydrazopyridinium‐associated states enables multiple recirculations of complementary carriers; the photoconductive gain *G* is proportional to the ratio of carrier lifetime (*τ*) to transit time (*τ*_tr_) [38].

**FIGURE 4:**
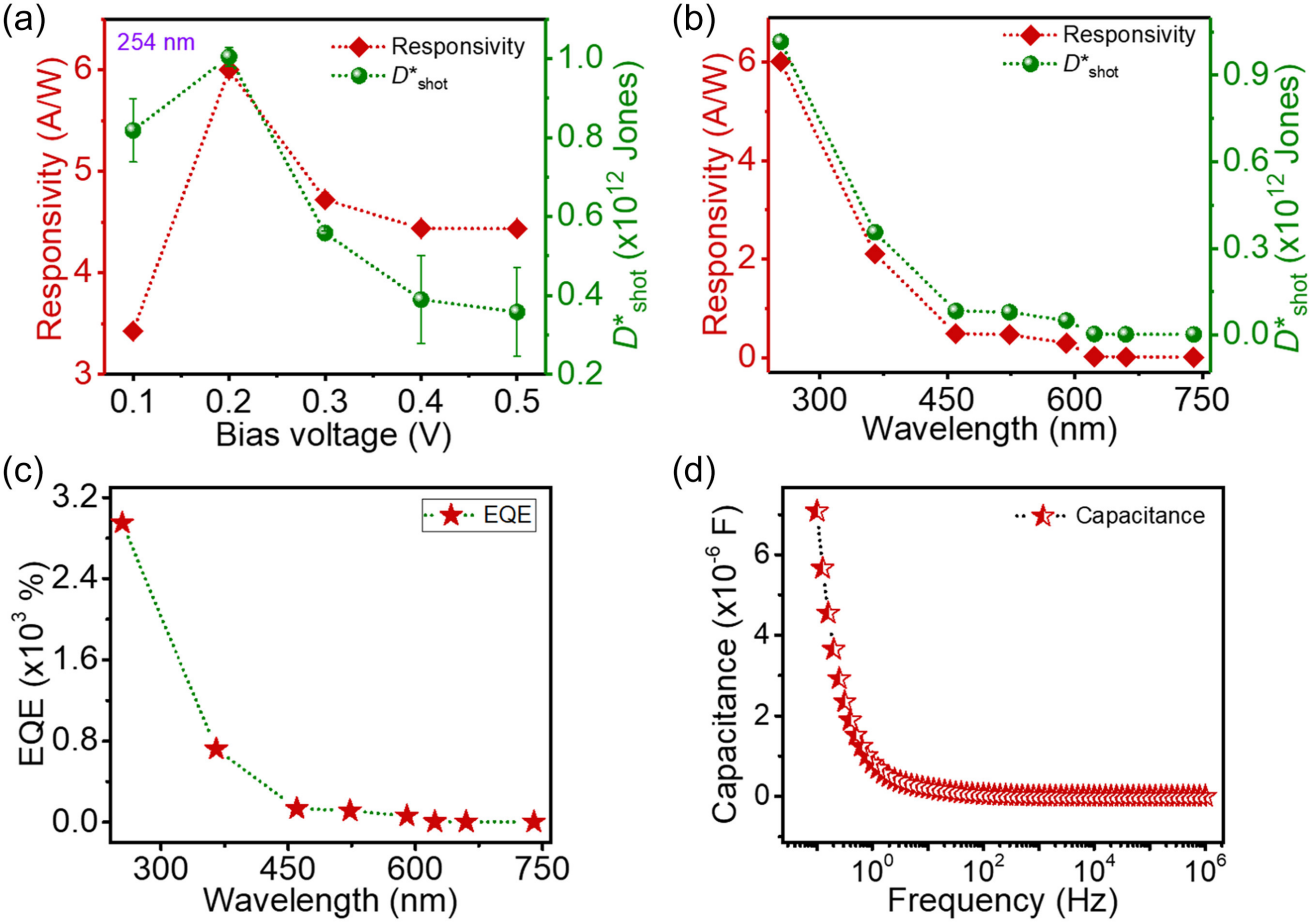
Photodetector performance metrics and gain mechanism of HPDLI. (a) Responsivity (*R*) and shot‐noise‐limited detectivity (*D* ^*^_shot_, mean ± s.d. across 5 devices; error bars for *R* are smaller than the symbol size) as a function of bias voltage (*V*_b_) under 254‐nm illumination (*P*_in_ = 80 µW/cm^2^). (b) Spectral response of *R* and *D* ^*^_shot_ as a function of wavelength at *V*_b_ = 0.2 V (*P*_in_ = 80 µW/cm^2^). (c) External quantum efficiency (EQE) as a function of wavelength (Vb = 0.2 V, Pin = 80 µW/cm^2^). (d) Frequency‐dependent capacitance (*C‐f*), measured in the dark from 0.1 Hz to 1 MHz at room temperature.

Spectral analysis revealed maximum performance at 254 nm for both *R* and *D* ^*^_shot_, with a systematic decline toward longer wavelengths (Figure 4b), consistent with the wavelength‐dependent absorption characteristics described above. The external quantum efficiency reached 2900% at 254 nm (Figure 4c), indicating that multiple carrier transits occur per absorbed photon. This behavior is attributed to photoconductive gain, in which long‐lived trapped carriers enable repeated transit of the complementary carrier type. This mechanism is well established in perovskite photodetectors, with reported EQE values ranging from hundreds to thousands of percent depending on trap state engineering and operating conditions [39]. The gain‐bandwidth product remains fundamentally limited by the carrier lifetime, explaining the tradeoff between high EQE and millisecond response times [38, 40].

To further elucidate the origin of the observed photoconductive gain, frequency‐dependent capacitance (*C‐f*) measurements were performed (Figure 4d). The device exhibits pronounced dielectric dispersion, with capacitance values increasing from ~10 pF at 1 MHz to 7 µF at 0.1 Hz. A single‐temperature frequency trace of this kind cannot by itself yield a validated electronic trap depth, which requires a temperature‐dependent emission analysis with a calibrated attempt frequency, nor can it separate electronic trapping from ionic motion, electrode polarization, or interfacial charge accumulation in a mixed ionic‐electronic conductor such as this [41]. Neither an absolute trap density (*N*_t_) nor a validated trap energy was quantified in this study; the capacitance dispersion is described only as evidence of a slow polarization process that may contribute to the photoconductive response, with temperature‐dependent admittance spectroscopy identified as the appropriate next step to disentangle the ionic and electronic contributions. Nonetheless, this low‐frequency dispersion, combined with the proposed interfacial band alignment (Figure S11 and Table S3), is consistent with the central structure–property relationship: the near‐linear Pb─I─Pb framework is consistent with the low optical gap and UV‐to‐visible response. The large apparent EQE is compatible with photoconductive gain; organic‐associated trapping is one proposed explanation, but its location and carrier dynamics have not been directly established.

Temporal stability measurements were recorded at ambient conditions of 25 ± 2 °C and 60%–65% RH. Under cyclic on/off photoswitching at 0.2 V and 254 nm, the on‐state photocurrent envelope shows a gradual decline over 2000s (Figure 5a). In contrast, continuous photoresponse measurements performed on the same device under uninterrupted illumination over the same 2000‐s duration exhibit negligible degradation, retaining approximately 98% of the initial photocurrent (evaluated by comparing the mean current over the final window at 1950–2000 s to the initial steady‐state plateau at 50–100 s; Figure 5b). This demonstrates robust operational durability under steady bias, whereas the envelope reduction in panel (a) primarily reflects dynamic contact or interfacial charge‐polarization effects associated with repeated switching rather than unrecoverable material degradation. Unencapsulated devices exposed to 80% relative humidity (RH) for 48 h show switching cycles of closely similar shape before and after exposure, normalized independently (Figure 5c), indicating retention of switching behavior rather than absolute photocurrent, dark current, or response time.

**FIGURE 5:**
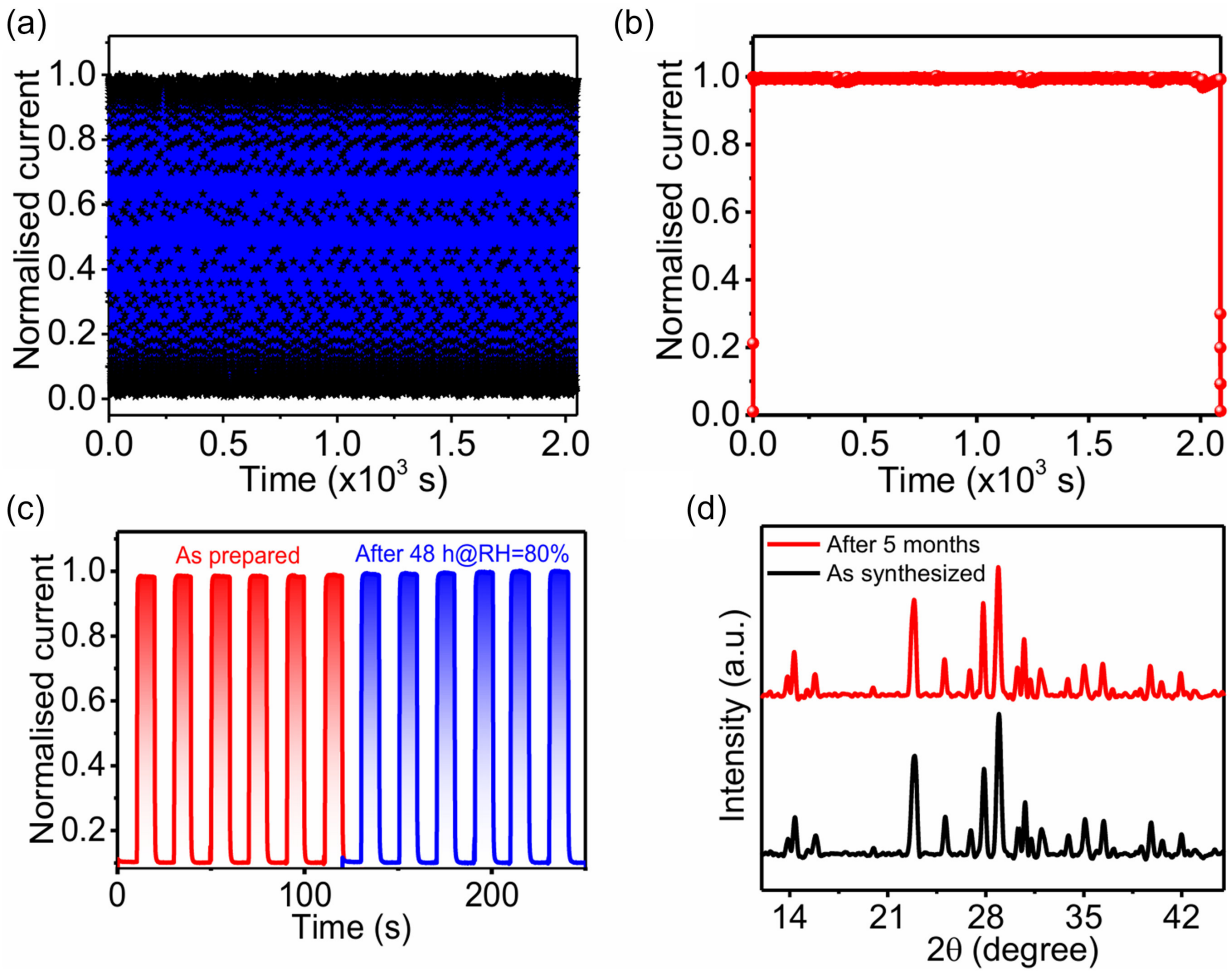
Preliminary operational, humidity, and structural stability screening of HPDLI. (a) Cyclic on/off switching over 2000s recorded under 254‐nm illumination (*P*_in_ = 80 µW/cm^2^) at 0.2 V bias. (b) Continuous photoresponse recorded on the same device under uninterrupted 254‐nm illumination over 2000s (*P*_in_ = 80 µW/cm^2^, Vb = 0.2 V), showing ~98% retention calculated between the initial stable state (e.g., 50–100 s) and final window (1950–2000s). (c) Dynamic on/off switching cycles recorded under 254‐nm illumination (*P*_in_ = 80 µW/cm^2^, *V*_b_ = 0.2 V) before (red) and after (blue) 48 h aging at 80% RH. (d) Powder X‐ray diffraction patterns of as‐synthesized HPDLI (black) and after 5 months of ambient storage (red).

Powder X‐ray diffraction analysis of HPDLI samples stored under ambient conditions for 5 months showed retention of the characteristic diffraction peak positions with no obvious additional crystalline peaks under the present measurement conditions (Figure 5d); because the traces are normalized and vertically offset, relative peak intensities are not used to quantify phase retention, and the comparison is limited to the peak position rather than intensity. Thermogravimetric analysis (TGA/‍DTG) shows a gradual mass loss of approximately 7%–8% before 350 °C, followed by two further mass‐loss regions and a residual mass of ~6.5% at 800 °C (Figure S16); this curve is reported descriptively rather than assigned to a specific decomposition pathway (Supporting Information). Bulky organic spacer layers have been reported to improve moisture resistance in some two‐dimensional perovskites; however, the hydrophobicity of the hydrazopyridinium spacer itself has not been directly assessed in this work (e.g., by contact‐angle or water‐vapor‐transmission measurements), so this ambient structural retention is reported as an observation rather than attributed to a confirmed moisture‐barrier mechanism [42]. This preliminary operational and humidity screening, together with the long‐term ambient structural retention observed without encapsulation, supports the potential suitability of HPDLI for practical optoelectronic deployment, with longer‐duration operational testing identified as an important next step.

Comparative analysis positions HPDLI among broadband perovskite photodetectors rather than UV‐selective devices. Li et al. [43] reported 198 mA/W responsivity at 377 nm for methylhydrazine lead bromide, Liu et al. [44] demonstrated high detectivity with MAPbI_3_‐MAPbBr_3_ heterojunctions, and Hu et al. [45] achieved 0.93 A/W with α‐FAPbI_3_; these devices operate primarily in the visible‐to‐near‐UV range. HPDLI extends photoresponse coverage to the deep‐UV region (254 nm) while maintaining a low operating voltage (0.2 V), 6 A/W responsivity, and photoconductive gain (2900% EQE), placing it among solution‐processed broadband photodetectors suited for low‐power operation [6, 39]. Potential applications include environmental monitoring, optical communication systems, and imaging configurations where spectral discrimination is achieved through external optical filtering [46, 47]. A comparison with both UV‐selective and broadband perovskite photodetectors is provided in Table S4.

## 3 Conclusion

Hydrazopyridinium lead iodide (HPDLI), a two‐dimensional Dion–Jacobson perovskite, illustrates a crystal engineering approach for achieving low‐bandgap photodetection in dimensionally confined systems. The orthorhombic crystal structure, featuring near‐linear Pb─I─Pb bonding (179.46°), yields a bandgap of 2.13 eV—among the lowest reported for *n = 1* 2D lead iodide systems—through enhanced orbital overlap between Pb 6s and I 5p states. Photodetectors achieve 6 A/W responsivity and 1.01 × 10^12^ Jones shot‐noise‐limited detectivity at 0.2 V bias, with 2900% external quantum efficiency consistent with trap‐mediated photoconductive gain and a proposed type‐II band alignment, neither of which has been directly measured. The broadband spectral response extends from deep‐UV through visible wavelengths, with higher absorption coefficients at shorter wavelengths providing enhanced UV sensitivity. Response times of 40/70 ms and ambient stability consistent with 2D perovskite architectures are compatible with applications in environmental monitoring, optical communications, and broadband imaging. These findings demonstrate that spacer‐controlled crystal symmetry can overcome the bandgap constraints imposed by octahedral tilting in *n = 1* lead iodide perovskites, enabling energy‐efficient, high‐gain photodetection.

## Funding

The Open Access Fees for this article was fully funded by the One Nation One Subscription (ONOS) Initiative of Govt. of India (https://ror.org/036h6g940). This study was supported by the CSIR‐NIIST DCPC Center of Excellence (PC‐II‐25014/3/2022‐PC II‐CPC, FTS: 3019123) is acknowledged. This work was also partially supported by the National Laboratories Scheme under the ULIP (Grant Number MLP002633). A.A., A.S., and A.V.D. acknowledge UGC, and C.M. acknowledges CSIR, for research fellowships. A.S. and K.S. acknowledge the Polish National Science Centre within the OPUS programme (grant agreement no. UMO‐2020/37/B/ST5/00663) and AGH University of Science and Technology within the program “Excellence Initiative–Research University,” as well as Polish high‐performance computing infrastructure PLGrid (HPC Center: ACK Cyfronet AGH) for computer facilities and support within computational grant no. PLG/2026/019667.

## Supporting information

Supplementary Material

## Data Availability

The data that support the findings of this study are available from the corresponding author upon reasonable request.
