# Supplementary material for: Two‐Dimensional Hydrazopyridinium Lead Iodide Perovskite Photodetectors With Broadband Sensitivity and Photoconductive Gain
Source: Small Sci. 2026 Oct 28;6(11):e70435. doi: 10.1002/smsc.70435 (PMC13715839; doi:10.1002/smsc.70435)
Supplement: Supplementary file 1 [file SMSC-6-e70435-s001.pdf]

Supporting Information

Two-Dimensional Hydrazopyridinium Lead Iodide Perovskite Photodetectors with Broadband Sensitivity and Photoconductive Gain

Avija Ajayakumar,[a,b] *Akash Suresh,* [a,b] *Andrzej Sławek,*[c] *Anandhu Gopan,*[a] *Chinnadurai Muthu,*[a,b] *Amarjith V. Dev,*[a,b] *Ashly Merlin Thomas,*[a] *Anusree Aravindakshan,*[a] *Konrad Szaciłowski,*[c,d] *and Chakkooth Vijayakumar*[*a,b]

[a] Chemical Sciences and Technology Division, CSIR-National Institute for Interdisciplinary Science and Technology (NIIST), Thiruvananthapuram 695 019, India

E-mail: vijay.niist@csir.res.in

[b] Academy of Scientific and Innovative Research (AcSIR), Ghaziabad 201 002, India

[c] Academic Centre for Materials and Nanotechnology, AGH University of Krakow, Mickiewicza 30, Krakow 30 059, Poland

[d] Unconventional Computing Lab, University of the West of England, Bristol BS16 1QY, United Kingdom

Experimental Section:

Chemicals: Lead (II) iodide (Sigma-Aldrich, 99.99 %), hydroiodic acid (Sigma-Aldrich, 57 wt% in H_2O), and 4,4’-Azopyridine (99.8 %) were used without further purification.

Single crystal synthesis: The HPDLI single crystals were synthesized by dissolving 46 mg of lead iodide (0.1 mmol) and 36 mg of 4,4’-azopyridine (0.2 mmol) in an aqueous solution of hydroiodic acid (HI) at 130 °C. The resulting hot yellow solution was gradually cooled to room temperature at a controlled rate of 5 °C/h, leading to the formation of deep red, flake-like crystals. The obtained crystals were subsequently washed with dichloromethane and dried under vacuum to remove residual solvents.

Photodetector device fabrication: A pre-patterned fluorine-doped tin oxide (FTO) transparent conductive glass substrate (1 cm × 1 cm) containing two electrodes separated by an inter-electrode channel length $L \approx 10$ μm and having an electrode-overlap width $W = 1.0$ cm was sequentially cleaned using acetone, deionized water, and isopropanol. Subsequently,

HPDLI (10 mg) was dissolved in N,N-dimethylformamide (DMF, 0.5 mL) to prepare the precursor solution. This solution was drop-cast across the channel and annealed at 90 °C for 30 minutes, yielding a film thickness of approximately 500 nm. The illuminated lateral-channel footprint, $A = L \times W \approx 1 \times 10^{-3}$ cm^2, rather than the full substrate area, was used in the device-metric calculations.

Characterization: The diffuse reflectance spectra of the HPDLI powder sample were recorded in the wavelength range of 200-800 nm using a Shimadzu UV-2600 UV-Vis spectrophotometer and transformed into the Kubelka-Munk remission function F(R), used as a proxy for the relative absorption/scattering behaviour of the powder sample rather than a calibrated thin-film absorption coefficient or ordinary transmission absorbance. Powder X-ray diffraction (PXRD) patterns of the perovskite crystals were obtained using a Xeuss Simultaneous 2D WAXS system with Cu-Kα radiation (λ = 1.5418 Å). Single-crystal X-ray diffraction data were collected at 300(2) K on a Bruker Kappa APEXII diffractometer. Data collection/cell refinement and reduction were performed with APEX4/SAINT and SAINT/XPREP, respectively. The structure was solved with SHELXT and refined against F^2 with SHELXL through WinGX; ORTEP3 was used for molecular graphics. Hydrogen atoms were placed geometrically and refined with constrained parameters (N–H atoms treated with AFIX 43). Scanning electron microscopy with energy-dispersive X-ray spectroscopy (SEM-EDS) was performed using a JEOL JSM-5600 LV microscope. Film thickness measurements were carried out using a Bruker Dektak XT stylus profilometer. Current-voltage (*I*-*V*) characteristics were recorded using a modular potentiostat/galvanostat (Vertex 100 mA/1A New Model, Ivium Technologies) at a scan rate of 100 mV/s. Ambient laboratory stability measurements were monitored at 25 ± 2 °C and 60-65% RH. For accelerated humidity aging, unencapsulated devices were housed in a sealed environmental chamber maintained at 80% RH (25 °C) for 48 h.

Electrochemical Impedance Spectroscopy (EIS): Electrical impedance and frequency-dependent capacitance were measured using a Solartron Analytical Materials Lab XM system over a frequency range of 1 Hz–1 MHz, using the reported AC perturbation amplitude of 0.1 V.

XPS, UPS, and LEIPS Characterization: Atomic concentrations were determined by X-ray photoelectron spectroscopy (XPS) on a PHI VersaProbe II Scanning XPS Microprobe using monochromatic Al Kα radiation (1486.6 eV) focused to a 100 μm spot. The photoelectron take-off angle was set to 66°, and the analyzer pass energy was 117.50 eV for survey scans. Chamber base pressure during analysis was maintained below 3×10^{-9} mbar.

Ultraviolet photoelectron spectroscopy (UPS) measurements were carried out in the same PHI VersaProbe II apparatus (ULVAC-PHI, Chigasaki, Japan) to determine the valence band maximum (VBM), ionization potential (IP), and work function (Φ). Spectra were excited using He I (21.22 eV) and He II (40.8 eV) resonance lines from an ultrahigh-vacuum gas discharge lamp. A sample bias of -5 V was applied to accelerate photoelectrons and clearly define the secondary electron cutoff (SE cutoff). The work function was evaluated from $\Phi = h\nu - (E_{\text{cutoff}} - E_F)$. Satellite emissions from secondary line excitations of the helium discharge were systematically subtracted incrementally, and exposure durations were minimized to avoid radiation damage.

Low-energy inverse photoemission spectroscopy (LEIPS) was employed to extract the conduction band minimum (CBM) and electron affinity (EA) utilizing low-energy incident electrons (< 5 eV). Near-UV optical emission from the sample surface was collected using a quartz lens and focused through band-pass filters (4.77 eV / 260 nm and 3.65 eV / 340 nm) prior to photon detection, ensuring the selected filter energy exceeded the EA of the material. Incident electron beam parameters were set to a current of 3 μA and an acceleration voltage of 40 V, with a step size of 0.02 V and a dwell time of 4000 ms per point. Energy onsets for both UPS and LEIPS were determined via linear tangent extrapolations at the inflection point intersecting the background baseline.

Use of AI-Assisted Writing Tools: Large language model (LLM)-based tools (ChatGPT; OpenAI; Sol version) were used to assist with language editing, formatting, and manuscript preparation during the course of this work. These tools were not used to generate scientific content, data, analysis, or conclusions. All intellectual contributions, experimental design, data interpretation, and scientific claims are solely the work of the authors, who have reviewed and take full responsibility for the content of the manuscript.

Use of AI-Generated Graphical Abstract: The Table of Contents/graphical abstract image accompanying this manuscript was generated using ChatGPT (OpenAI; Sol version) from a text prompt describing the reported chemistry, crystal structure, and photodetector performance, supplied by the authors. The image is a stylized, conceptual illustration: it does not depict, represent, or replace any experimental data, spectrum, micrograph, or measurement, and no figure of research data in the manuscript or this Supporting Information was created, altered, or enhanced using AI. All authors reviewed the image for scientific accuracy and appropriateness prior to submission.

Computational methodology: Density Functional Theory (DFT)[1] simulations of the electronic structure of the solid-state HPDLI was performed with CASTEP software[2] in

order to calculate the electronic band structure. We used the generalized gradient approximation (GGA) with the Perdew-Burke-Ernzerhof (PBE) functional, a semilocal exchange-correlation functional. CASTEP-implemented on-the-fly generated ultrasoft pseudopotentials were used with a plane wave kinetic energy cut-off set to 517 eV (fine quality). Van der Waals interactions were included via the semi-empirical Tkatchenko-Scheffler (TS) dispersion correction. Prior to the band structure calculations, the experimental crystal structure of HPDLI was geometry-optimized. The separation of Monkhorst-Pack k-point grid was set to 0.01 $Å^{-1}$. Geometry optimization and electron spectroscopy calculations were considered convergent when the energy change per atom was less than 10^{-5} and 10^{-6} eV, respectively. Gaussian-like Fermi smearing was used. The distance cutoff point for bond populations was set to 3.0 Å. Spin polarization and explicit spin-orbit coupling were not included in these calculations.

Calculation of polyhedral distortion of PbI_6^{4-}:

To quantitatively evaluate the local polyhedral distortion of the PbI_6^{4-} inorganic framework, the octahedral quadratic elongation (λ) and polyhedral angle variance (σ^2) were calculated according to the method of Robinson et al.[3]:

$$\lambda = \frac{1}{6}\sum_{i=1}^{6}\left(\frac{l_i}{l_0}\right)^2$$

$$\sigma^2 = \frac{1}{11}\sum_{i=1}^{12}(\theta_i - 90°)^2$$

where l_i and θ_i represent the individual Pb–I bond lengths and *cis*-I–Pb–I bond angles, respectively, and l_0 is the bond length of a regular octahedron of equal volume. Using the six refined Pb–I distances (3.1305–3.3049 Å), the six-iodine coordination polyhedron has a convex-hull volume of 44.24 $Å^3$, giving l_0 = 3.2133 Å; the calculated values are a quadratic elongation of λ=1.0117 and an angle variance of σ^2 =40.48 deg^2. These parameters describe intra-octahedral bond-length and angular spread and are distinct from the near-linear inter-octahedral Pb–I–Pb bridging angle (179.46°), which governs metal-halide orbital overlap at the band edge.

Calculation of device parameters:

Responsivity (*R*): *R* is the ratio of the photocurrent to P_{in} and can be calculated as

$R = I_{ph}/(P_{in} \times A)$

and $I_{ph} = I_{light} - I_{dark}$

where I_{ph}, I_{light}, I_{dark}, P_{in}, and A are the photocurrent, current under light illumination, dark current, incident light intensity, and the effective device area, respectively.

Detectivity (D^*_{shot}) reflects the ability of PDs to detect weak light signals. D^*_{shot} is directly proportional to R, and inversely proportional to the square root of I_{dark}. D^*_{shot} can be estimated using the following equation:

$$D^*_{shot} = A^{1/2} \times R/(2e \times I_{dark})^{1/2}$$

where e is the elementary charge. This is a conventional dark-current-based theoretical estimate that treats shot noise from dark current as the only noise source. A directly measured dark-current noise spectral density was not available for this work, so D^*_{shot} is reported as an idealized shot-noise-limited estimate rather than a directly measured noise-limited detectivity. This device is a photoconductor operating with gain; for such devices a unity-gain shot-noise model of this kind can overestimate the achievable detectivity relative to a gain-aware noise model that also accounts for the photoconductive gain and an excess noise factor. A gain-aware correction was not calculated here because the required noise-relevant parameters (a measured current-noise spectral density and the excess noise factor) are not available. The reported value should accordingly be read as an idealized theoretical estimate rather than a validated sensitivity figure; the practical value may be lower if Johnson, generation–recombination, contact, 1/f, or gain-related excess noise contributes.

External quantum efficiency (EQE) can be defined as the efficiency of PDs to convert incident photons to current, or simply the ratio between the output carriers and the number of incident photons. EQE can be given as:

$$\text{EQE} = Rhc/e\lambda$$

where h, c, e, and λ are the Planck constant, speed of light, electron charge, and wavelength of incident light, respectively.

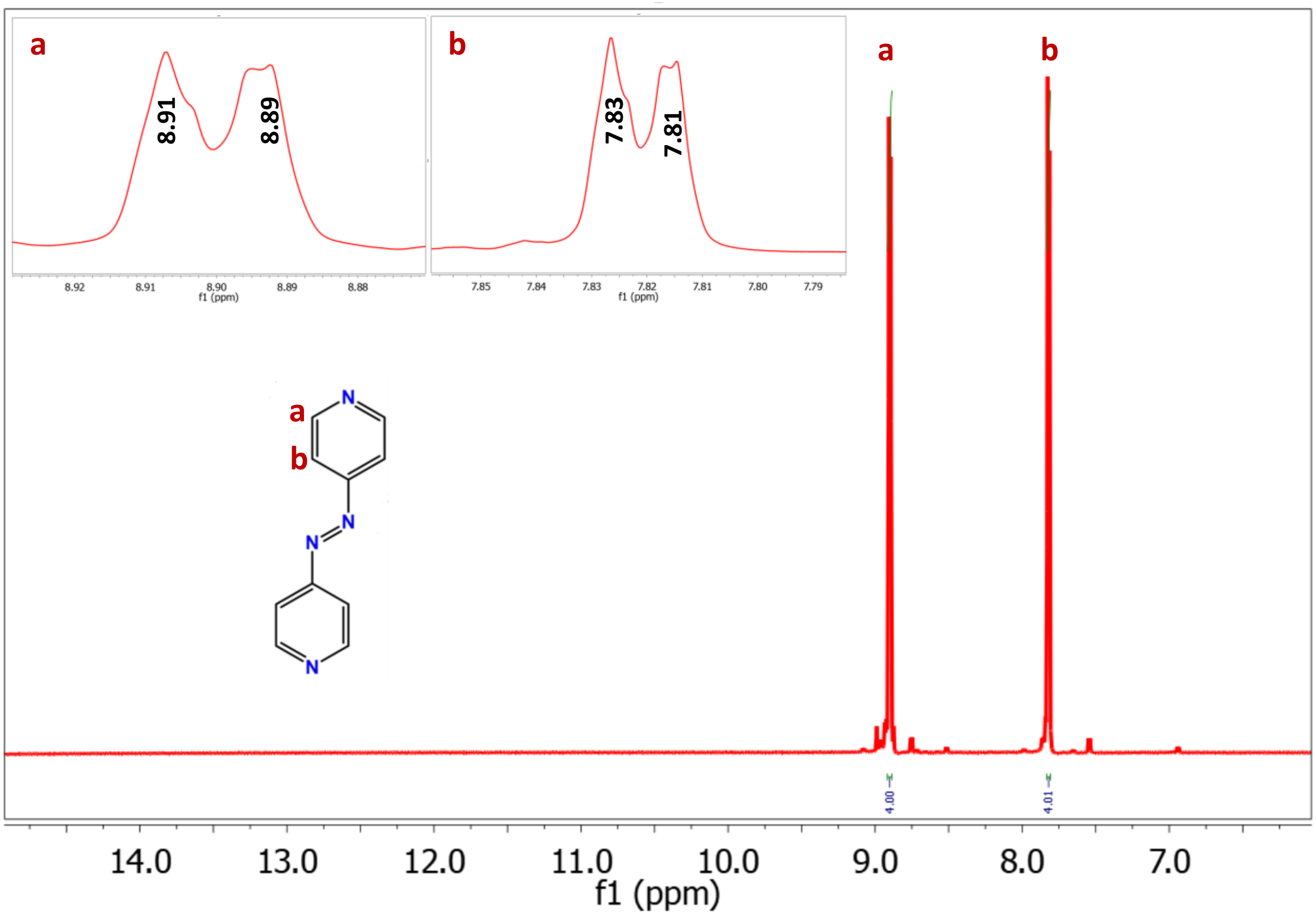

Figure S1: ^{1}H-NMR spectrum (DMSO-d_6) of the 4,4'-azopyridine starting precursor. The insets display zoomed-in views of the aromatic doublets corresponding to protons H_a (δ = 8.91 ppm) adjacent to the pyridine nitrogen atoms and protons H_b (δ = 7.83 ppm) adjacent to the central azo (–N=N–) linkage.

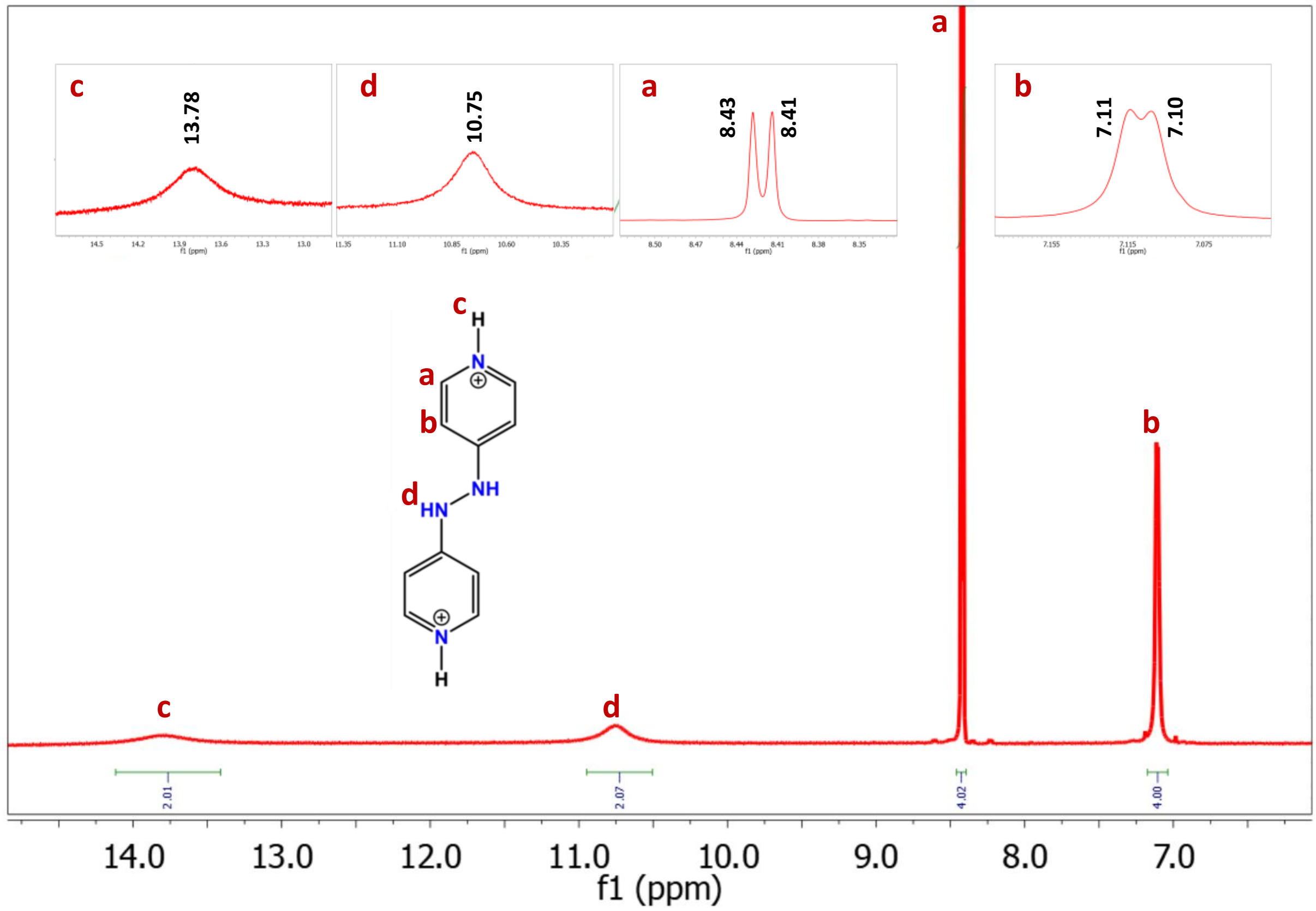

Figure S2: ^{1}H-NMR spectrum (DMSO-d_6) of dissolved HPDLI single crystals confirming the *in situ* reduction of 4,4'-azopyridine to the divalent hydrazopyridinium cation (HPD). The insets display zoomed-in views of the broad pyridinium –NH^+ resonance (H_c, δ = 13.78 ppm), the broad hydrazo –NH–NH– resonance (H_d, δ = 10.75 ppm), the aromatic doublet (H_a, δ = 8.43 ppm) adjacent to the pyridinium nitrogen atoms, and the aromatic doublet (H_b, δ = 7.11 ppm) adjacent to the hydrazo linkage.

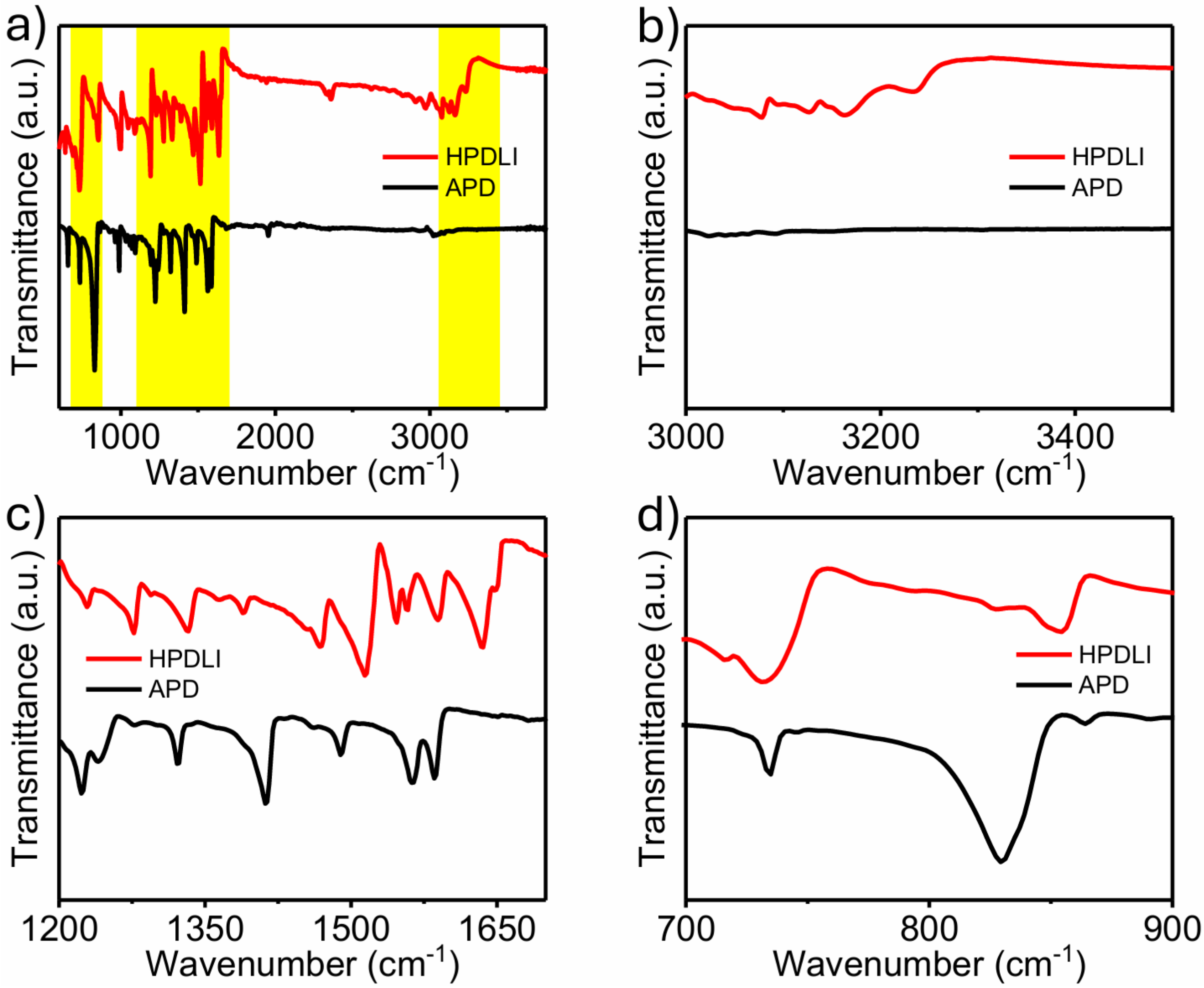

Figure S3: FTIR spectra comparing the precursor ligand 4,4'-azopyridine (APD; black) and the HPDLI perovskite single crystals (red) confirming the *in situ* structural transformation during synthesis. a) Full survey spectra highlighting the key vibrational regions (700-3500 cm^{-1}). b) Zoomed-in high-wavenumber region (3000-3500 cm^{-1}) showing broad –NH– stretching modes in HPDLI associated with hydrazopyridinium and pyridinium protonation. c) Mid-wavenumber fingerprint region (1200-1700 cm^{-1}) showing shifts in aromatic C = C/C = N ring modes and central linkage vibrations. d) Low-wavenumber region (700-900 cm^{-1}) displaying characteristic out-of-plane C – H bending modes of the pyridinium ring.

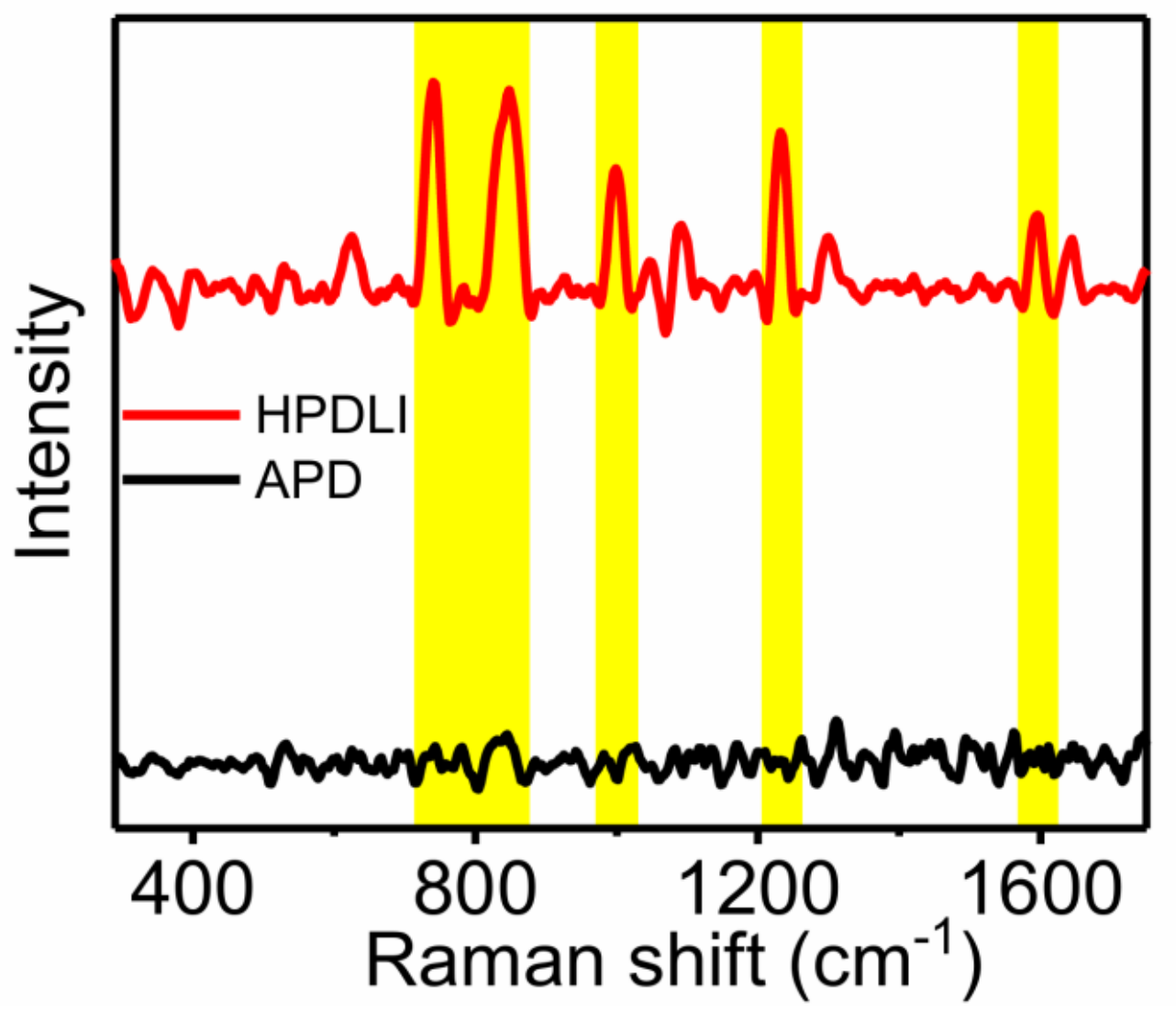

Figure S4: Comparative Raman spectra of the 4,4'-azopyridine precursor ligand (APD; black) and HPDLI perovskite single crystals (red) measured in the range of 300-1700 cm^{-1}. The highlighted yellow bands indicate prominent vibrational modes in HPDLI, including bands at 750-850 cm^{-1}), aromatic ring deformation and C – H bending modes (1000 cm^{-1}), the central hydrazo N – N single-bond stretching vibration (1250 cm^{-1}), and the pyridinium ring C = C/C = N stretching modes (1600 cm^{-1}).

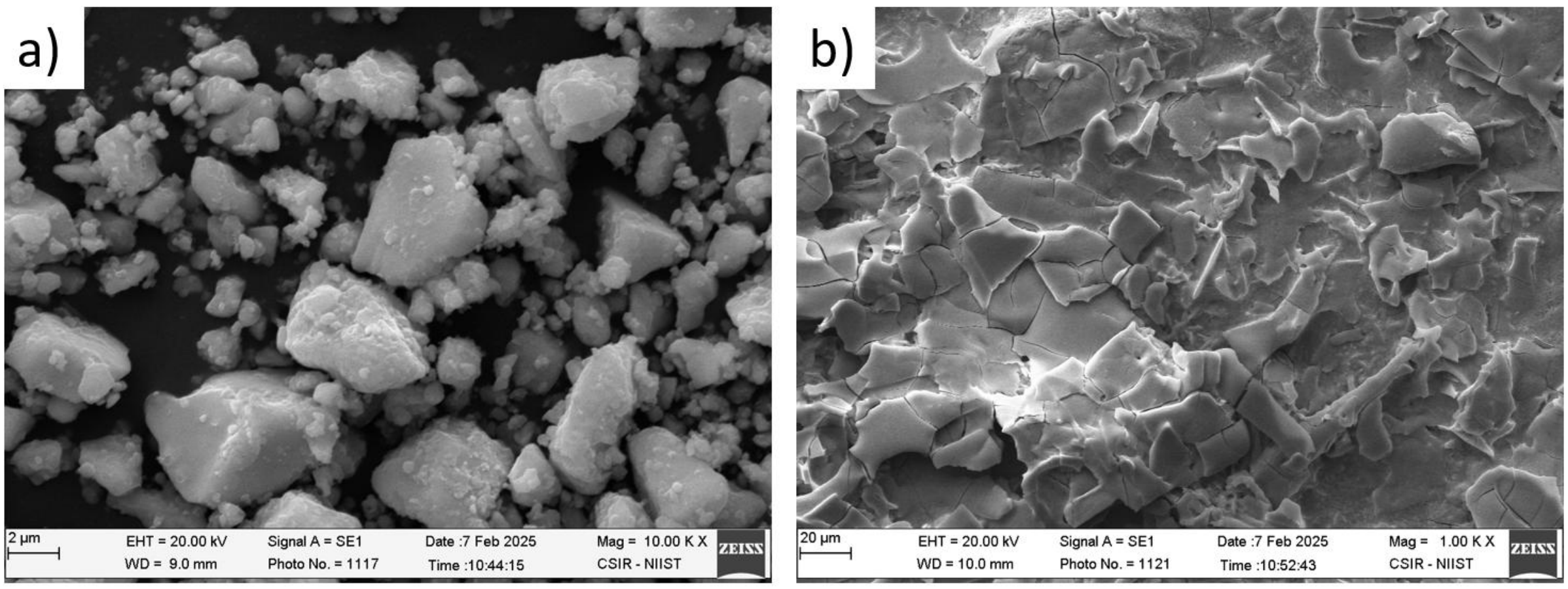

Figure S5: SEM images showing surface morphology of HPDLI: a) Powder sample; b) Drop-cast thin film on FTO substrate. The layered morphology characteristic of two-dimensional perovskites is clearly visible in both forms, consistent with the long-range periodicity determined from single-crystal X-ray diffraction. The uniform film coverage on the FTO substrate is observed across the imaged area.

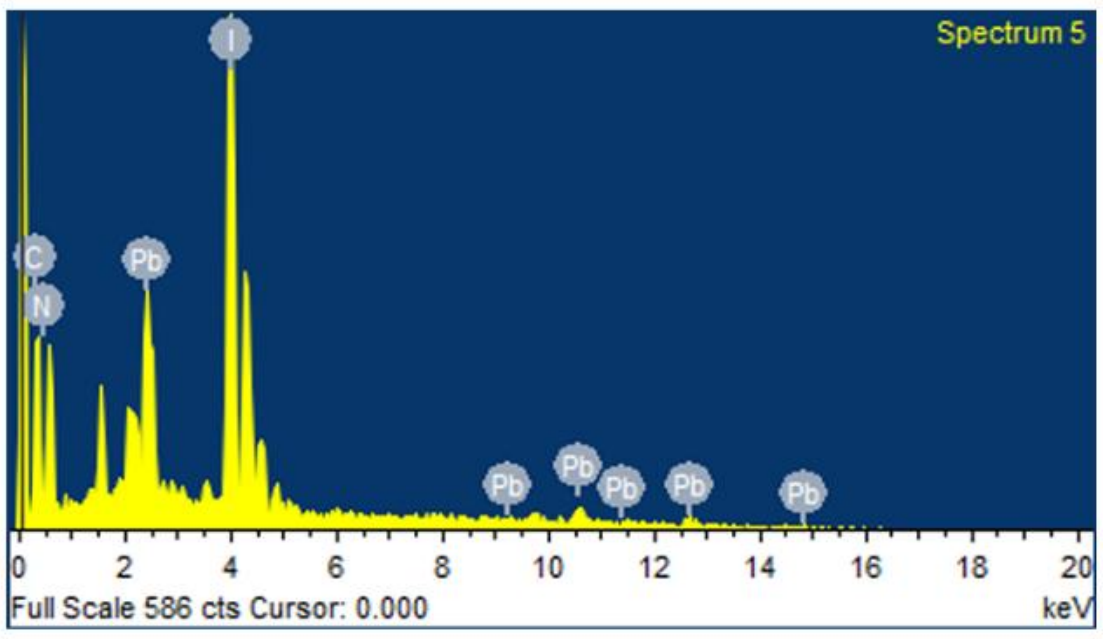

Element	Weight %	Atomic %
C K	17.71	56.66
N K	8.42	23.09
I L	55.84	16.90
Pb M	18.03	3.34

Figure S6: Energy Dispersive X-ray Spectroscopy (EDS) analysis of HPDLI detecting the expected constituent elements (Pb, I, N, C) for the single analyzed sample/area shown. Composition is treated as semiquantitative given the known limitations of EDS for light elements (C, N) and for the heavy/light element pair I/Pb, and is not used here to independently confirm the SCXRD-derived stoichiometry.

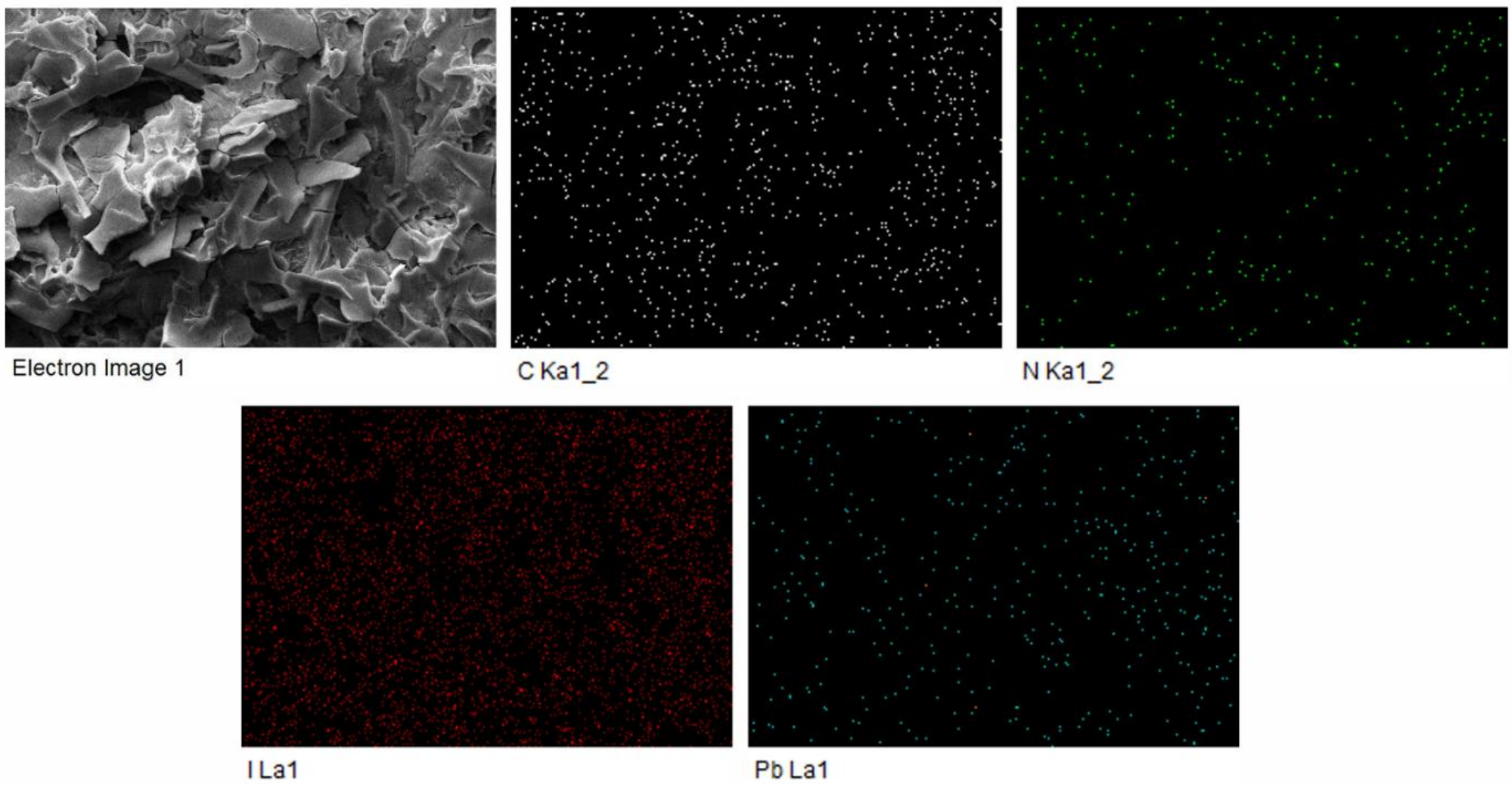

Figure S7: Elemental mapping images of HPDLI revealing the spatial distribution of constituent elements (Pb, I, N, C). A uniform distribution of all elements is observed within the imaged field of view shown; this is not used to infer whole-sample uniformity.

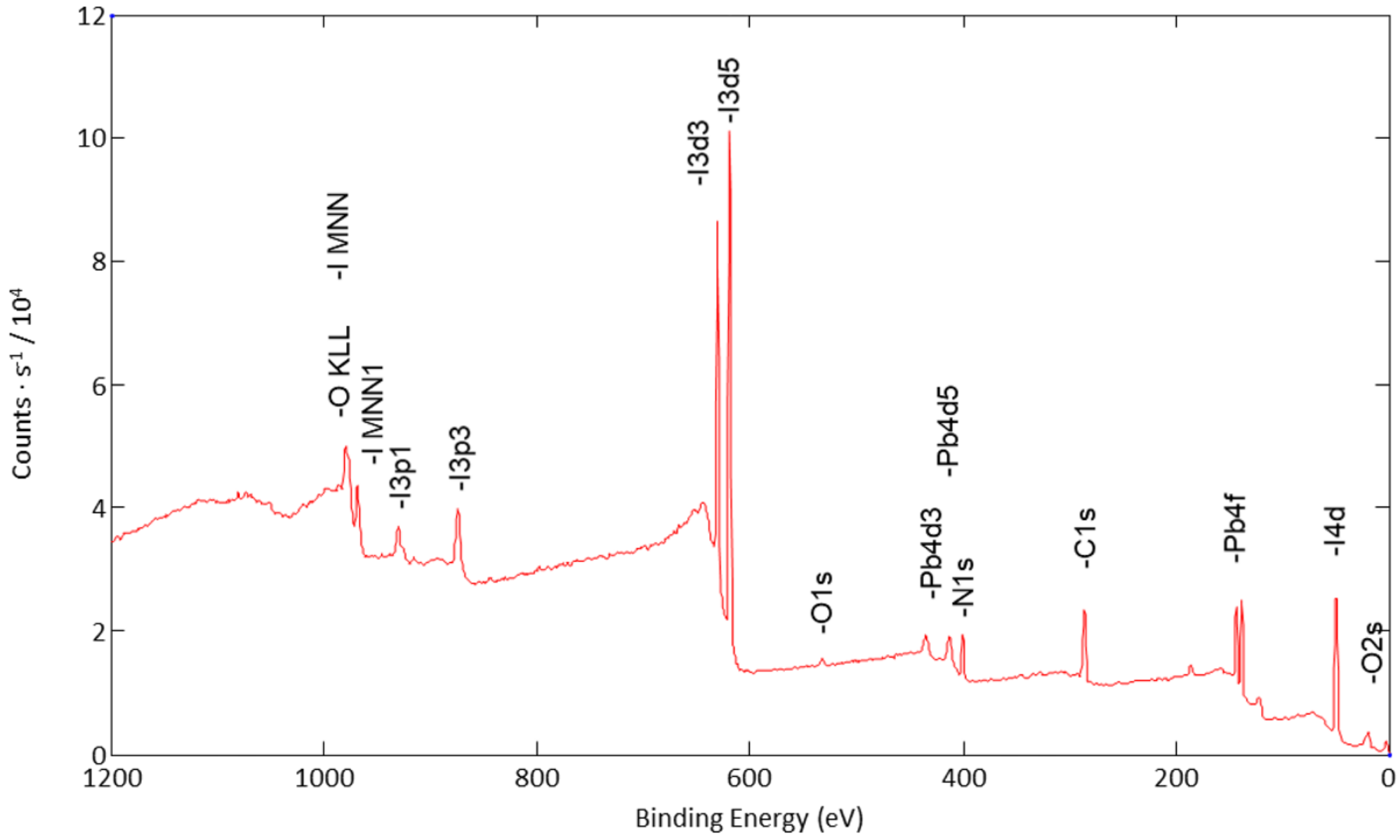

Figure S8: Wide-scan X-ray photoelectron spectroscopy (XPS) survey spectrum of the HPDLI film showing characteristic core levels (I 3*d*, Pb 4*f*, C 1*s*, and N 1*s*), showing the detected survey-level elemental signals under the stated acquisition conditions; this measurement supports the surface elemental assignment but is not used by itself to establish crystallographic phase purity.

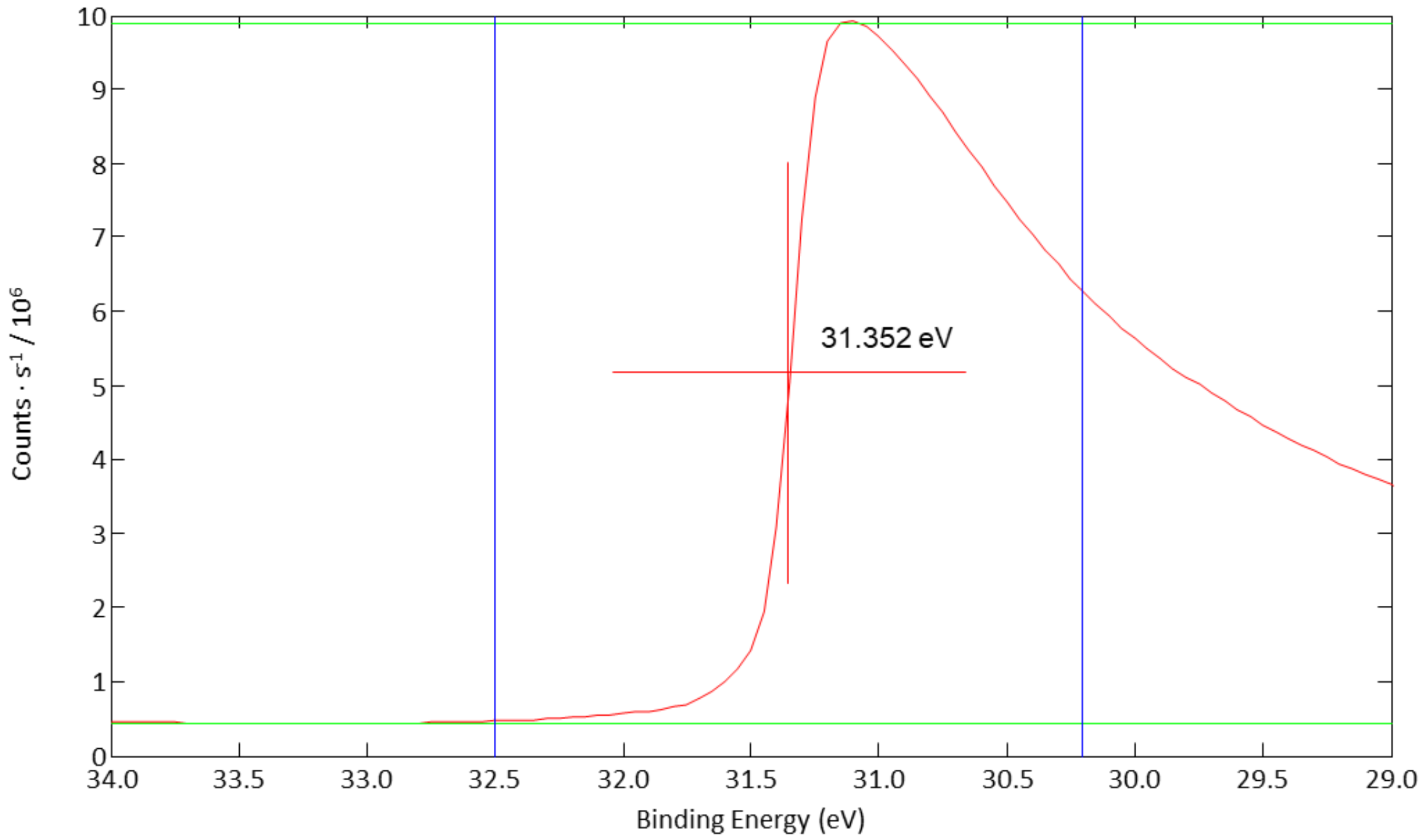

Figure S9: Ultraviolet photoelectron spectroscopy (UPS) secondary-electron cut-off (SECO) spectrum of the HPDLI film, yielding a cut-off energy of 31.352 eV and a corresponding work function of Φ = 4.5 eV.

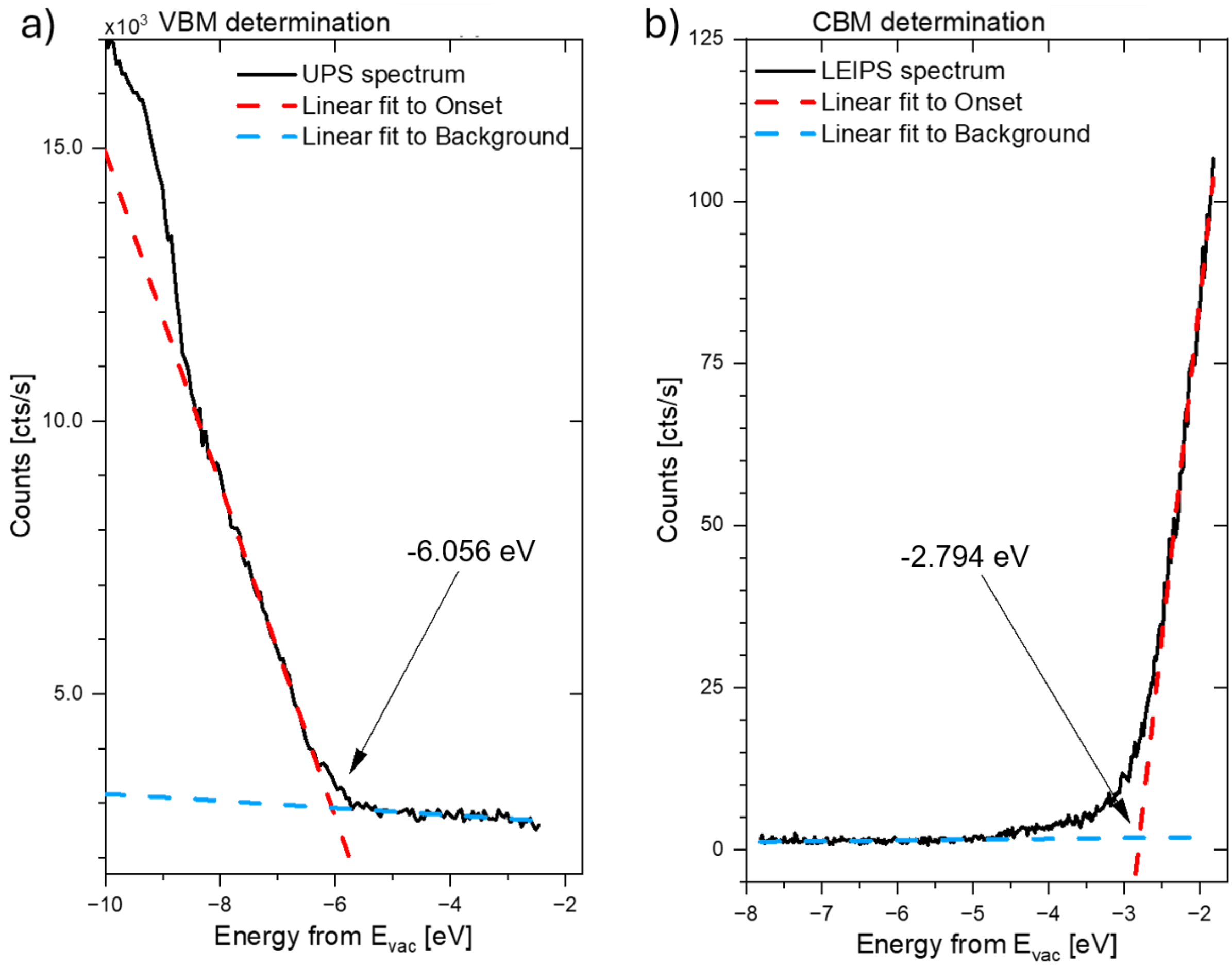

Figure S10: (a) UPS valence band spectrum with linear extrapolation of the leading edge determining the valence band maximum (VBM = -6.056 eV / ionization potential = 6.056 eV relative to vacuum). (b) Low-energy inverse photoemission spectroscopy (LEIPS) conduction band spectrum with linear onset fit determining the conduction band minimum (CBM = -2.794 eV/ electron affinity = 2.794 eV relative to vacuum; VBM/CBM and IP/EA are reported here to the full precision of the fitted onsets, consistent with Table S3 and Figure S11).

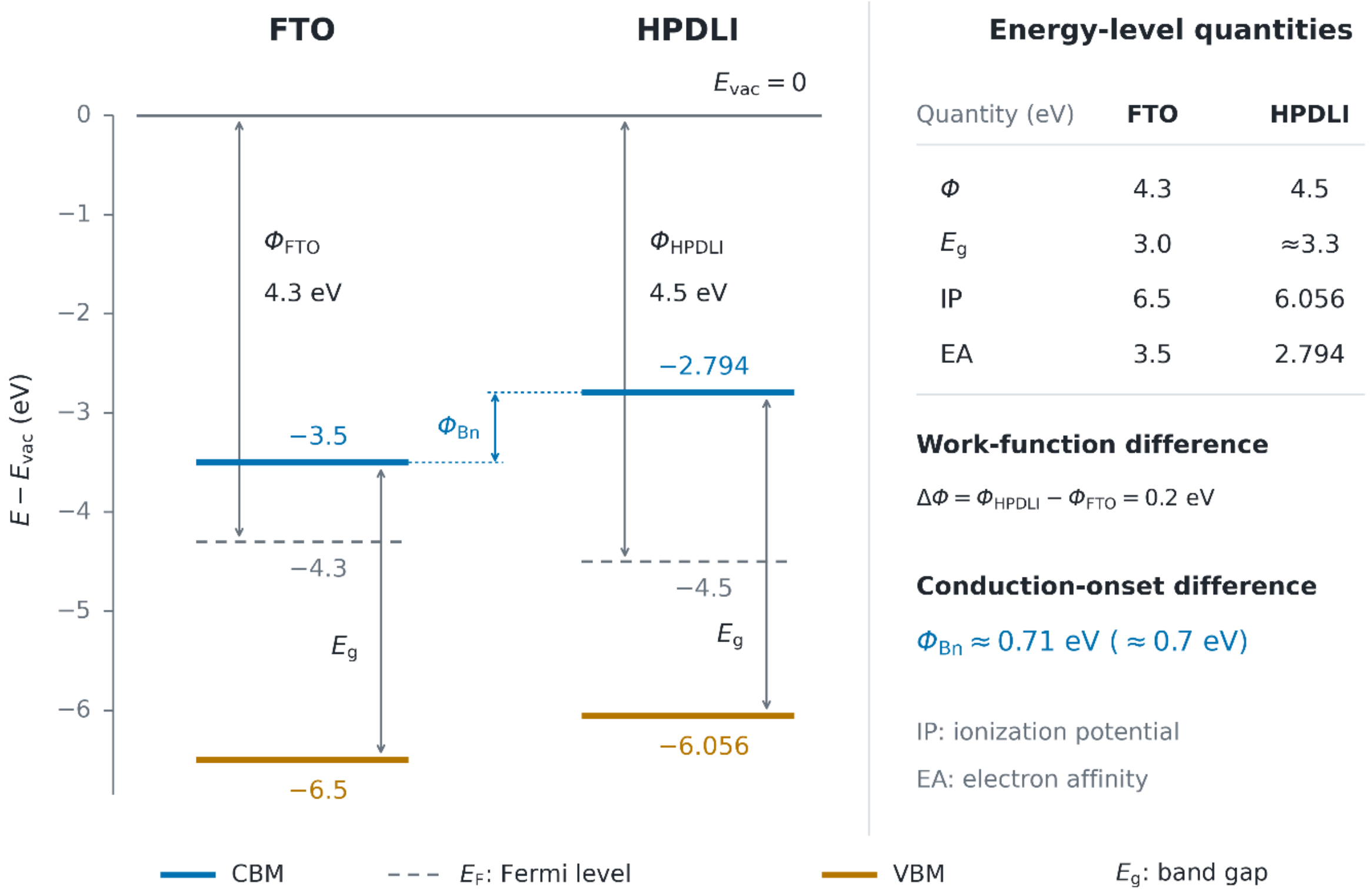

Figure S11: Energy-level comparison of FTO and HPDLI. Schematic diagram constructed from UPS and LEIPS data, with energies referenced to a common vacuum level. The work-function difference is ($\Delta\Phi$ = 0.2 eV), and the difference between the listed conduction-band onsets, denoted (ΔE_C), is approximately 0.71 eV (≈0.7 eV); this onset difference is not itself a measured Schottky injection barrier (ΦBn). This energy-level comparison does not, by itself, establish an interfacial dipole or a kinetically operative electron-injection barrier.

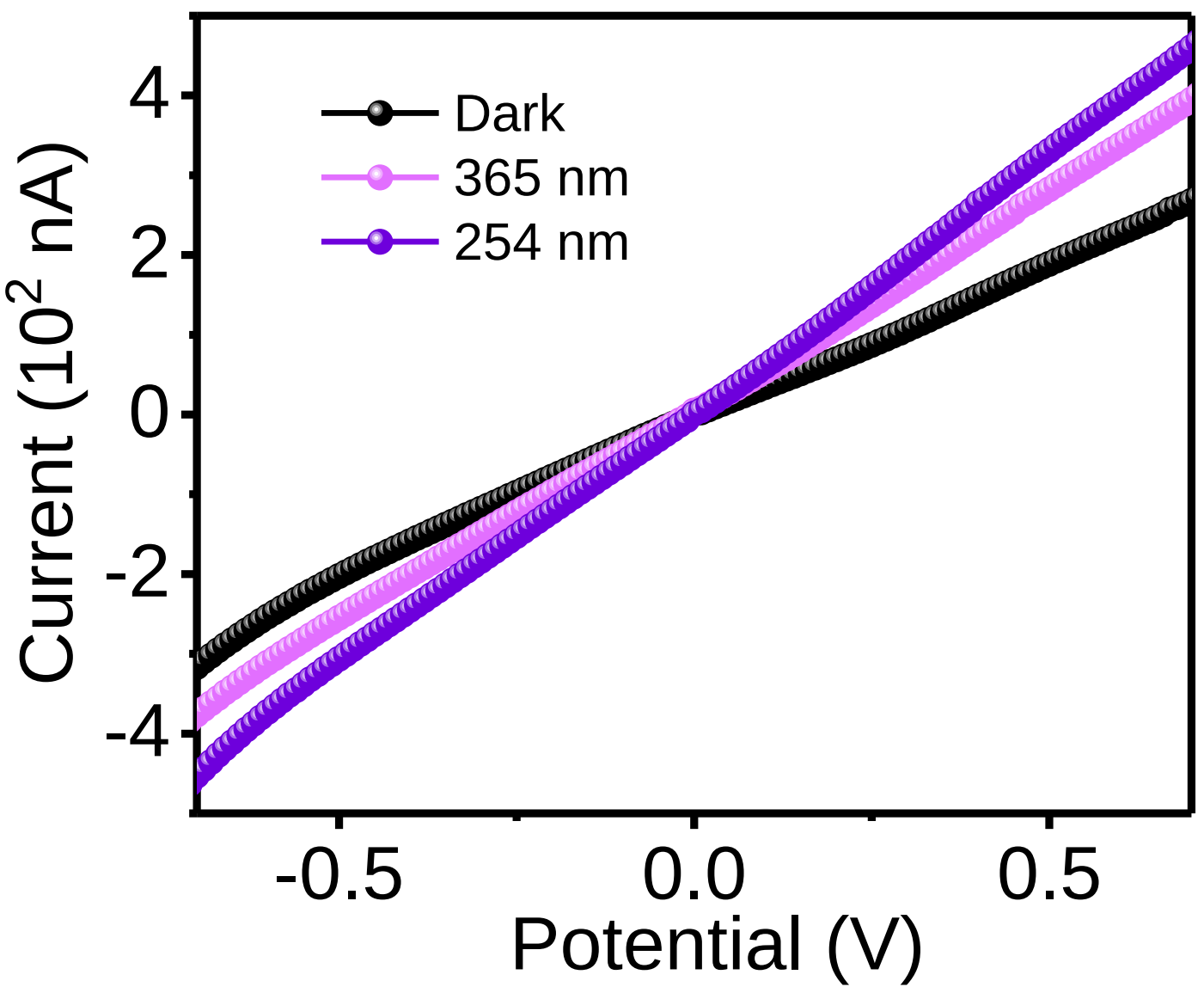

Figure S12: Signed, linear-scale current–voltage (*I*-*V*) characteristics of the lateral FTO/HPDLI/FTO photodetector under dark conditions and UV illumination at 254 nm (P_{in} = 80 μW/cm^2) and 365 nm (P_{in} = 140 μW/cm^2) swept between -0.7 V and + 0.7 V, demonstrating approximately symmetric conduction in the measured voltage range (symmetric contacts can produce symmetric I-V behaviour even in the presence of injection barriers).

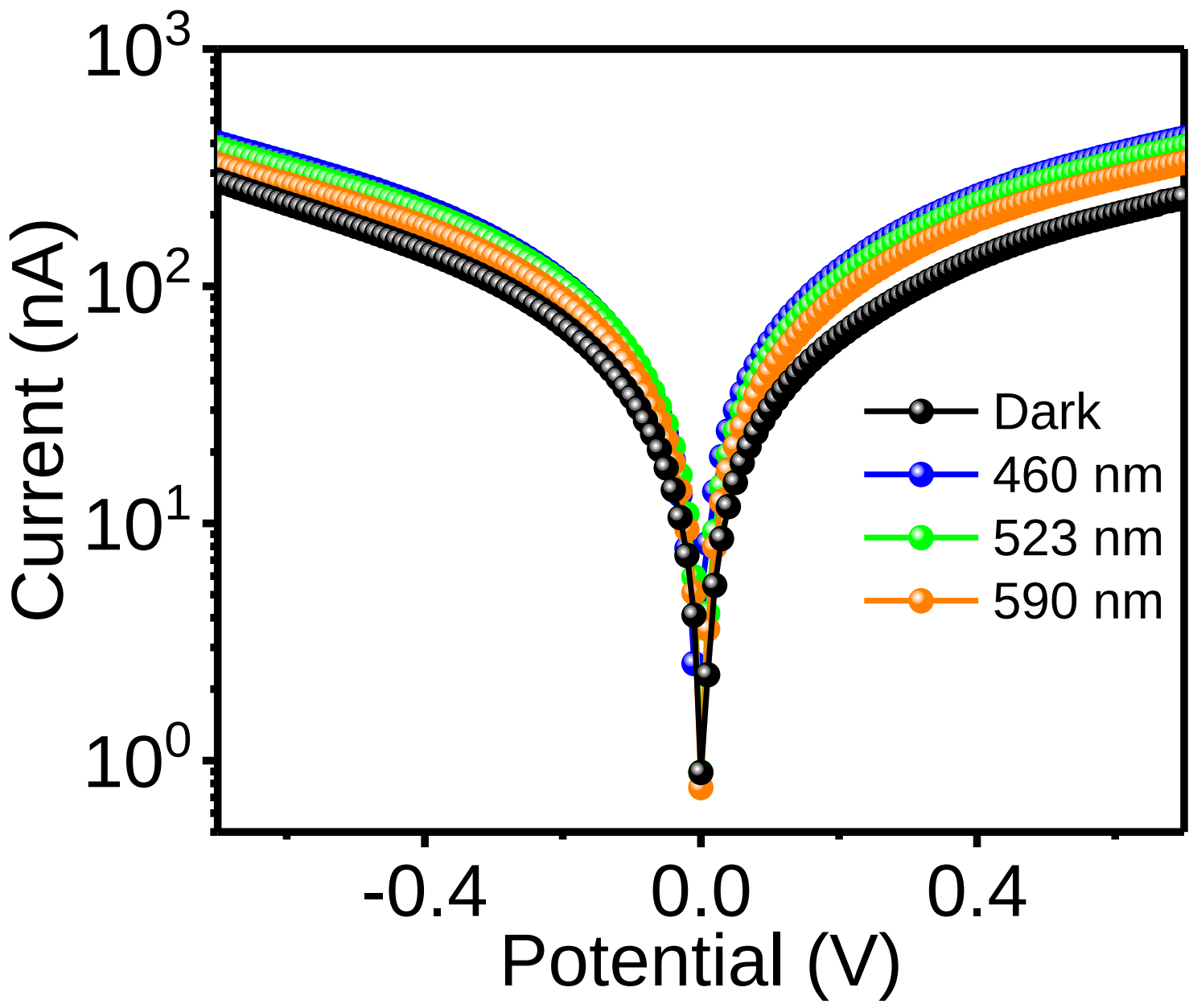

Figure S13: Current–voltage (*I*-*V*) characteristics of the lateral HPDLI photodetector in the dark (black) and under visible light illumination at 460 nm (blue), 523 nm (green), and 590

nm (orange) at a matched incident optical power density of P_{in} = 80 μW/cm^2, plotted on a semi-logarithmic scale from -0.7 V to+0.7 V.

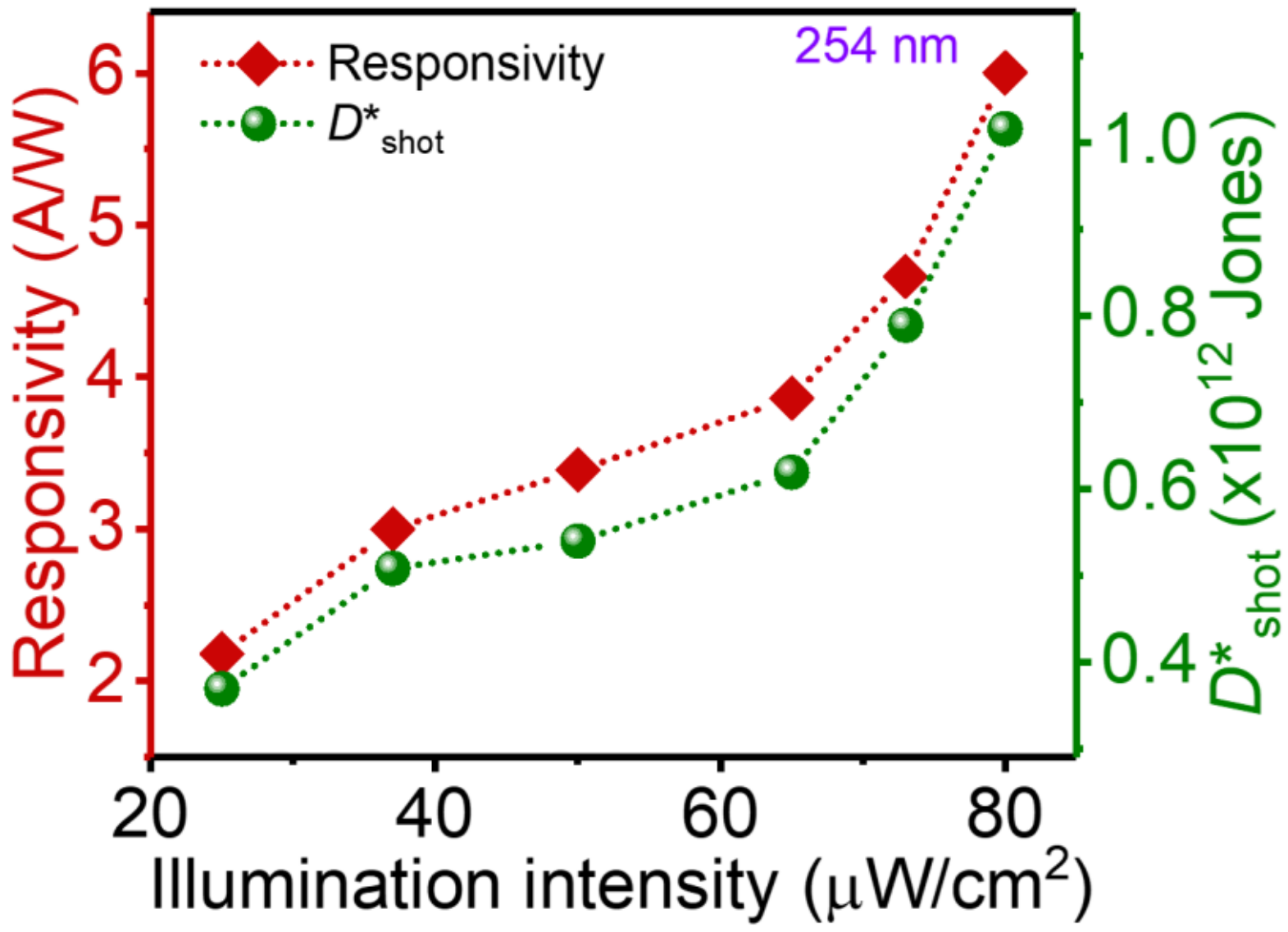

Figure S14: Responsivity (R) and shot-noise-limited detectivity (D^*_{shot}) as a function of incident power (P_{in} = 25-80 μW/cm^2) at V_b = 0.2 V.

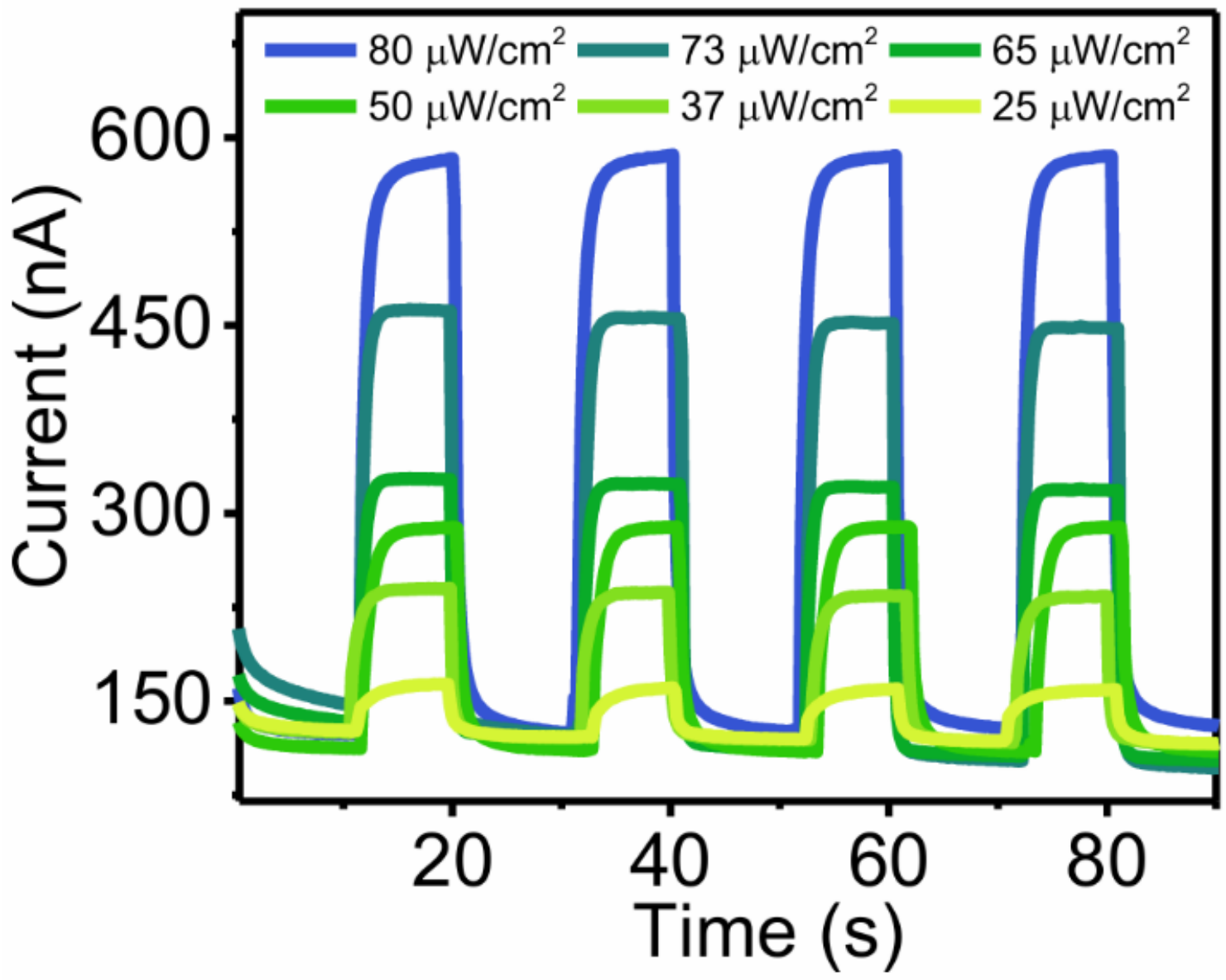

Figure S15: Illumination intensity (P_{in}) dependent photoresponse showing systematic increase in photocurrent with incident power under 254 nm illumination at an applied bias of 0.2 V(from yellow to blue curves: P_{in} = 25, 37, 50, 65, 73, and 80 μW cm^{-2}). The ~3.2-fold intensity span tested is too narrow to extract a reliable power-law exponent or to establish a specific trap-activation mechanism; a substantially wider intensity range would be needed for either. The evaluated incident irradiance (25-80 μW cm^{-2}) was selected within the calibrated

linear irradiance window of the deep-UV optical test enclosure to ensure stable signal detection while aiming to minimize the risk of local heating; active-layer temperature was not measured. The longer settling observed at 80 µW cm^{-2} relative to 73 µW cm^{-2} occurred in a single trace at each intensity; these data cannot distinguish trap filling from source stabilization, device history or drift, photothermal effects, or ionic/contact relaxation, so no unique mechanism is assigned.

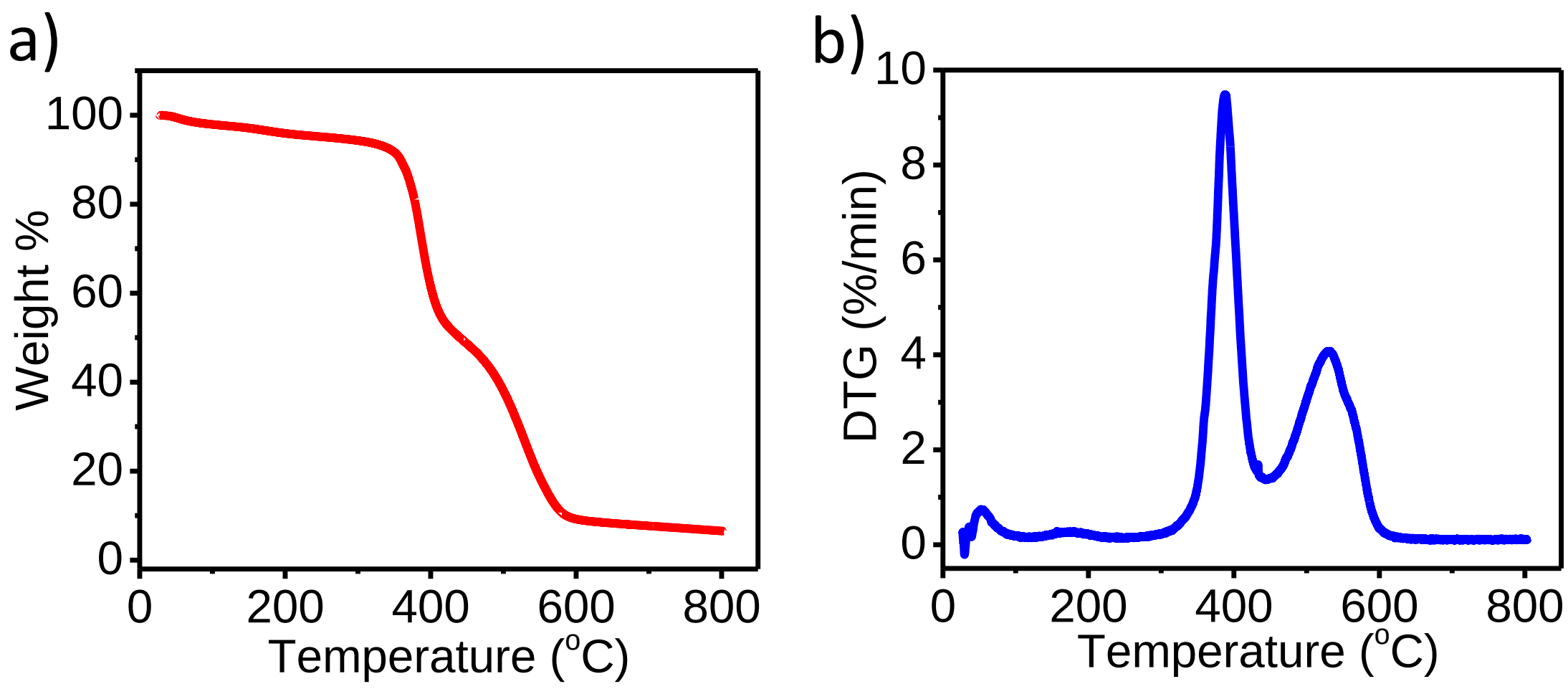

Figure S16: Thermal stability analysis of HPDLI single crystals recorded from room temperature to 800 °C under an inert nitrogen atmosphere at a heating rate of 10 °C/min. a) Thermogravimetric analysis (TGA) curve showing a gradual mass loss of approximately 7–8% before 350 °C, followed by two further mass-loss regions. b) Derivative thermogravimetric (DTG) curve showing mass-loss maxima near 390 °C and 535 °C; observation-only, without assignment to a specific decomposition pathway (see below).

The residual mass at 800 °C is approximately 6.5 % of the initial sample weight. We report this curve descriptively rather than assigning the mass-loss regions uniquely to loss of the organic dication followed by a specific lead-iodide residue: without evolved-gas analysis (e.g., TGA-MS, as used for related lead-iodide perovskites[4]) or characterization of the final residue, such an assignment is not yet demonstrated for this material, and we do not invoke a methylammonium lead iodide decomposition-pattern analogy in its place.

Table S1. Comparison of crystal systems between the previously reported structure with this work.

	Reported crystal	This work
Crystal System, space group	Monoclinic, P2/c	Orthorhombic, Pbcm
Unit cell dimension, *a*, *b*, *c*	*a* 12.1123(12) Å	*a* 6.4148(2) Å
	b 6.4042(7) Å	*b* 12.8547(5) Å
	c 12.8373(13) Å	*c* 23.3069(9) Å
Cell angles	α=γ= 90°, β=106.058°	α=β=γ= 90°
Cell volume	956.93 (17) $Å^3$	1921.89 $Å^3$
R-factor	10.19%	1.85%

The unit cell dimensions reflect different crystallographic descriptions, with our orthorhombic setting containing twice the number of formula units compared to the previous monoclinic description, rather than representing fundamental structural divergence. A key metric in single-crystal X-ray crystallography, the *R* value (residual factor), quantifies the agreement between the observed structure factor amplitudes (F_{obs}) and the calculated structure factor amplitudes (F_{calc}) derived from the proposed atomic model. This parameter is critical for assessing the accuracy and reliability of the structural refinement. A lower *R* value signifies a better fit between the experimental diffraction data and the refined model, whereas an elevated *R* value may indicate systematic errors in data collection, improper modelling, or unresolved structural disorder. The present refinement gives $R1[I > 2\sigma(I)] = 0.0185$ (1.85 %) and $wR2(\text{all}) = 0.0423$, compared with $R = 0.1019$ (10.19 %) for the previously reported monoclinic structure. The lower *R*1 value indicates closer agreement between the observed diffraction data and the refined structural model under the stated measurement and refinement conditions; it is not, by itself, interpreted as evidence for a lower bulk defect density or higher sample crystallinity.

Table S2. Comparison of structural family (RP: Ruddlesden–Popper; DJ: Dion–Jacobson), in-plane Pb-I-Pb bond angles, and optical bandgaps (E_g) for representative n=1 2D layered lead iodide perovskites reported in the literature.

Sl. No.	2D Perovskite	Structural Family	Pb-I-Pb angle (°)	Bandgap (eV)	Ref.
1	(Naphthalene-O-propyl-$NH_3)_2PbI_4$	RP	146.869	2.98	5
2	$(2\text{-Br-PEA})_2PbI_4$	RP	144.54	2.8	6
3	$(4\text{-Br-PMA})_2PbI_4$	RP	153.042	2.74	7
4	$(4\text{-Cl-PMA})_2PbI_4$	RP	154.49	2.73	7
5	$(NEA)_2PbI_4$	RP	154.15	2.7	8
6	$(4\text{-F-PMA})_2PbI_4$	RP	158.343	2.69	7
7	$(PMA)_2PbI_4$	RP	158.42	2.66	7,9
8	$(4\text{-I-PMA})_2PbI_4$	RP	156.112	2.66	7
9	$(C_4H_3SC_2H_4NH_3)_2PbI_4$	RP	152.73	2.66	10
10	$(EDBE)PbI_4$	DJ	165.01	2.638	11,12
11	$(2\text{-F-PEA})_2PbI_4$	RP	150.1	2.62	6
12	$(2\text{-Cl-PEA})_2PbI_4$	RP	151.22	2.61	6
13	$(NH_3C_8H_{16}NH_3)PbI_4$	DJ		2.58	13
14	$(BAESBT)_2PbI_4$	RP	150.635	2.535	14
15	$(PA)_2PbI_4$	RP	153.68	2.47	15,16
16	$(PEA)_2PbI_4$	RP	153.08	2.46	17
17	$(BA)_2PbI_4$	RP	155.20	2.43	18,19
18	$(NH_3C_7H_{14}NH_3)PbI_4$	DJ		2.43	13
19	$(NH_3C_9H_{18}NH_3)PbI_4$	DJ	153.75	2.43	13
20	$(PDMA)PbI_4$	DJ		2.42	20
21	$(m\text{-PDA})PbI_4$	DJ	143.8	2.42	21
22	$(HA)_2PbI_4$	RP	155.65	2.41	15
23	$(4,4\text{-DFCHA})_2PbI_4$	RP	155.671	2.38	22
24	$(4AMP)PbI_4$	DJ	154.325	2.38	23
25	$(EOA)_2PbI_4$	RP	159.93	2.35	24
26	$(3AMPY)PbI_4$	DJ	164.52	2.34	25

27	$(CHA)_2PbI_4$	RP	155.912	2.332	26
28	$(4,4\text{-}DFHHA)_2PbI_4$	RP	173.34	2.32	27
29	$(DPDA)PbI_4$	DJ	171.849	2.29	28
30	$(DFPD)_2PbI_4$	RP	169.97	2.24	29
31	$(3AMP)PbI_4$	DJ	166.115	2.23	23
32	$(MHy)_2PbI_4$	RP	175.15	2.21	30
33	$(APD)PbI_4$	DJ	180	2.19	31
34	HPDLI	DJ	179.46	2.13	This work

Entries 13, 18, and 20 lack reported Pb–I–Pb bond angles and were omitted from the linear correlation analysis (N = 31)

Table S3. Summary of experimental electronic parameters, energetic band edge positions (VBM and CBM), electronic bandgap (E_g), ionization potential (IP), and electron affinity (EA) for HPDLI and the pre-patterned FTO substrate determined from UPS and LEIPS measurements.

Material	Work Function Φ (eV)	VBM (eV)	CBM (eV)	Electronic Gap E_g (eV)	Ionization Potential (eV)	Electron Affinity (eV)
HPDLI	4.5	-6.056	-2.794	3.26	6.056	2.794
FTO	4.3	-6.50	-3.50	3.00	6.5	3.5

As UPS and LEIPS are inherently surface-sensitive probes (sampling only the outermost few nanometers) that do not resolve organic-versus-inorganic orbital character, the 3.26 eV electronic gap in Table S3 is treated as a surface-accessible transport gap: it is not used as a direct, bulk-equivalent measure of the optical (Tauc, 2.13 eV) or DFT (1.936 eV) gaps discussed in the main text, is not used to infer an exciton binding energy from the difference, and does not by itself validate the proposed type-II organic/inorganic band alignment; see Section 2 of the main manuscript for the fuller discussion.

Table S4. Selected lead-halide-perovskite photodetectors (UV-selective and broadband). Reported responsivity and detectivity values are reproduced under the test conditions and

noise assumptions stated in the cited sources and are not necessarily directly comparable; where a source calculates detectivity from dark-current shot noise rather than a measured noise spectrum, the value is identified as D^{*}shot. For references 39 and 40, the reported values correspond to a CsPbBr3 nanocrystal/quantum-dot down-conversion (colour-converting) layer combined with a separate underlying photodiode, not a monolithic CsPbBr3 photodetector; detectivity values for all comparison entries are reproduced as reported in the cited sources and were not independently re-derived.

Material	Responsivity (mA/W)	Detectivity (× 10^{11} Jones)	Wavelength (nm)	Bias Voltage (V)	Ref.
$CsPbBr_3$ QDs	146	1.54	365	20	32
$CsPbBr_3$ film	50	0.882	365	20	32
$CsPbBr_3$	0.24	0.011	254	2	33
$(FAPbCl_3)_{1-x}(MAPbCl_3)_x$	5.6	4.03	254	0	34
$(FAPbI_3)_{1-x}(MAPbBr_3)_x$	4.92	0.75	254	0	35
$MAPbBr_3$	4.57	102	254	2	36
$(C_8H_{11}FN)_2PbBr_4$	288	1.6	254	10	37
Glass/$CsPbBr_3$ nanosheet/Au	85	4.05	265	3	38
$CsPbBr_3$	84	74	270	5	39
$CsPbBr_3$ QDs/CPI film/ITO glass/$MAPbI_3$/Au	1.4	2.4	279	0.1	40
$MHyPbBr_3$	198	142	377	0	41
$(TPA3)_2PbBr_4$	156	1.26	405	7	42
HPDLI	6×10^3	10.1 (D^{*}_{shot})	254	0.2	This work

References

1. W. Kohn, A. D. Becke, R. G. Parr, "Density Functional Theory of Electronic Structure," *The Journal of Physical Chemistry* 100 (1996): 12974–12980, https://doi.org/10.1021/jp960669l.
2. S. J. Clark, M. D. Segall, C. J. Pickard, et al., "First principles methods using CASTEP," *Zeitschrift für Kristallographie - Crystalline Materials* 220 (2005): 567–570, https://doi.org/10.1524/zkri.220.5.567.65075.
3. K. Robinson, G. V. Gibbs, P. H. Ribbe, "Quadratic Elongation: A Quantitative Measure of Distortion in Coordination Polyhedra," *Science* 172 (1971): 567–570, https://doi.org/10.1126/science.172.3983.567.
4. E. J. Juarez-Perez, Z. Hawash, S. R. Raga, L. K. Ono, Y. Qi, "Thermal degradation of CH3NH3PbI3 perovskite into NH3 and CH3I gases observed by coupled thermogravimetry–mass spectrometry analysis," *Energy & Environmental Science* 9 (2016): 3406–3410, https://doi.org/10.1039/C6EE02016J.
5. J. V. Passarelli, D. J. Fairfield, N. A. Sather, et al., "Enhanced Out-of-Plane Conductivity and Photovoltaic Performance in n = 1 Layered Perovskites through Organic Cation Design," *Journal of the American Chemical Society* 140 (2018): 7313–7323, https://doi.org/10.1021/jacs.8b03659.
6. D. B. Straus, S. Hurtado Parra, N. Iotov, et al., "Tailoring Hot Exciton Dynamics in 2D Hybrid Perovskites through Cation Modification," *ACS Nano* 14 (2020): 3621–3629, https://doi.org/10.1021/acsnano.0c00037.
7. M. H. Tremblay, J. Bacsa, B. Zhao, et al., "Structures of $(4\text{-Y-}C_6H_4CH_2NH_3)_2PbI_4$ {Y = H, F, Cl, Br, I}: Tuning of Hybrid Organic Inorganic Perovskite Structures from Ruddlesden–Popper to Dion–Jacobson Limits," *Chemistry of Materials* 31 (2019): 6145–6153, https://doi.org/10.1021/acs.chemmater.9b01564.
8. K. Du, Q. Tu, X. Zhang, et al., "Two-Dimensional Lead(II) Halide-Based Hybrid Perovskites Templated by Acene Alkylamines: Crystal Structures, Optical Properties, and Piezoelectricity," *Inorganic Chemistry* 56 (2017): 9291–9302, https://doi.org/10.1021/acs.inorgchem.7b01094.
9. L. Mao, H. Tsai, W. Nie, et al., "Role of Organic Counterion in Lead- and Tin-Based Two-Dimensional Semiconducting Iodide Perovskites and Application in Planar Solar Cells," *Chemistry of Materials* 28 (2016): 7781–7792, https://doi.org/10.1021/acs.chemmater.6b03054.
10. H. Dammak, S. Elleuch, H. Feki, et al., "Synthesis, Crystal Structure, Vibrational Spectra, Optical Properties and Theoretical Investigation of a Two-Dimensional Self Assembled

Organic-Inorganic Hybrid Material," *Solid State Sciences* 61 (2016): 1–8, https://doi.org/10.1016/j.solidstatesciences.2016.08.014.

11. E. R. Dohner, A. Jaffe, L. R. Bradshaw, et al., "Intrinsic White-Light Emission from Layered Hybrid Perovskites," *Journal of the American Chemical Society* 136 (2014): 13154–13157, https://doi.org/10.1021/ja507086b.
12. D. Cortecchia, S. Neutzner, A. R. Srimath Kandada, et al., "Broadband Emission in Two-Dimensional Hybrid Perovskites: The Role of Structural Deformation," *Journal of the American Chemical Society* 139 (2017): 39–42, https://doi.org/10.1021/jacs.6b10390.
13. X. Li, J. Hoffman, W. Ke, et al., "Two-Dimensional Halide Perovskites Incorporating Straight Chain Symmetric Diammonium Ions, $(NH_3C_mH_{2m}NH_3)(CH_3NH_3)_{n-1}Pb_nI_{3n+1}$ (m = 4-9; n = 1-4)," *Journal of the American Chemical Society* 140 (2018): 12226–12238, https://doi.org/10.1021/jacs.8b07712.
14. X.-H. Zhu, N. Mercier, P. Frère, et al., "Effect of Mono- versus Di-Ammonium Cation of 2,2′-Bithiophene Derivatives on the Structure of Organic−Inorganic Hybrid Materials Based on Iodo Metallates," *Inorganic Chemistry* 42 (2003): 5330–5339, https://doi.org/10.1021/ic034235y.
15. I. Spanopoulos, I. Hadar, W. Ke, et al., "Uniaxial Expansion of the 2D Ruddlesden−Popper Perovskite Family for Improved Environmental Stability," *Journal of the American Chemical Society* 141 (2019): 5518–5534, https://doi.org/10.1021/jacs.9b01327.
16. D. G. Billing, A. Lemmerer, "Synthesis, Characterization and Phase Transitions in the Inorganic–Organic Layered Perovskite-Type Hybrids $[(C_nH_{2n+1}NH_3)_2PbI_4]$, n = 4, 5 and 6," *Acta Crystallographica Section B: Structural Science* 63 (2007): 735–747, https://doi.org/10.1107/S0108768107031758.
17. J. Yu, J. Kong, W. Hao, et al., "Broadband Extrinsic Self-Trapped Exciton Emission in Sn-Doped 2D Lead-Halide Perovskites," *Advanced Materials* 31 (2019): 1806385, https://doi.org/10.1002/adma.201806385.
18. D. B. Mitzi, "Synthesis, Crystal Structure, and Optical and Thermal Properties of $(C_4H_9NH_3)_2MI_4$ (M = Ge, Sn, Pb)," Chemistry of Materials 8 (1996): 791–800, https://doi.org/10.1021/cm9505097.
19. C. C. Stoumpos, D. H. Cao, D. J. Clark, et al., "Ruddlesden–Popper Hybrid Lead Iodide Perovskite 2D Homologous Semiconductors," *Chemistry of Materials* 28 (2016): 2852–2867, https://doi.org/10.1021/acs.chemmater.6b00847.

20. Y. Li, J. V. Milić, A. Ummadisingu, et al., "Bifunctional Organic Spacers for Formamidinium-Based Hybrid Dion–Jacobson Two-Dimensional Perovskite Solar Cells," *Nano Letters* 19 (2019): 150–157, https://doi.org/10.1021/acs.nanolett.8b03552.
21. L. Gao, X. Li, B. Traoré, et al., "m-Phenylenediammonium as a New Spacer for Dion–Jacobson Two-Dimensional Perovskites," *Journal of the American Chemical Society* 143 (2021): 12063–12073, https://doi.org/10.1021/jacs.1c03687.
22. T. T. Sha, Y. A. Xiong, Q. Pan, et al., "Fluorinated 2D Lead Iodide Perovskite Ferroelectrics," *Advanced Materials* 31 (2019): 1901843, https://doi.org/10.1002/adma.201901843.
23. L. Mao, W. Ke, L. Pedesseau, et al., "Hybrid Dion–Jacobson 2D Lead Iodide Perovskites," *Journal of the American Chemical Society* 140 (2018): 3775–3783, https://doi.org/10.1021/jacs.8b00542.
24. X. Chen, H. Lu, K. Wang, et al., "Tuning Spin-Polarized Lifetime in Two-Dimensional Metal–Halide Perovskite through Exciton Binding Energy," *Journal of the American Chemical Society* 143 (2021): 19438–19445, https://doi.org/10.1021/jacs.1c08514.
25. X. Li, W. Ke, B. Traore, et al., "Two-Dimensional Dion−Jacobson Hybrid Lead Iodide Perovskites with Aromatic Diammonium Cations," *Journal of the American Chemical Society* 141 (2019): 12880–12890, https://doi.org/10.1021/jacs.9b06398.
26. H. Y. Ye, W. Q. Liao, C. L. Hu, et al., "Bandgap Engineering of Lead-Halide Perovskite-Type Ferroelectrics," *Advanced Materials* 28 (2016): 2579–2586, https://doi.org/10.1002/adma.201505224.
27. X. G. Chen, X. J. Song, Z. X. Zhang, et al., "Confinement-Driven Ferroelectricity in a Two-Dimensional Hybrid Lead Iodide Perovskite," *Journal of the American Chemical Society* 142 (2020): 10212–10218, https://doi.org/10.1021/jacs.0c03710.
28. M. P. Hautzinger, J. Dai, Y. Ji, et al., "Two-Dimensional Lead Halide Perovskites Templated by a Conjugated Asymmetric Diammonium," *Inorganic Chemistry* 56 (2017): 14991–14998, https://doi.org/10.1021/acs.inorgchem.7b02285.
29. D. Kim, B. Dryzhakov, Y. Liu, et al., "Ferroelectric and Charge Transport Properties in Strain-Engineered Two-Dimensional Lead Iodide Perovskites," *Chemistry of Materials* 33 (2021): 4077–4088, https://doi.org/10.1021/acs.chemmater.1c00679.
30. M. Mączka, M. Ptak, A. Gągor, et al., "Layered Lead Iodide of [Methylhydrazinium]$_2$PbI$_4$ with a Reduced Band Gap: Thermochromic Luminescence and Switchable Dielectric Properties Triggered by Structural Phase Transitions," *Chemistry of Materials* 31 (2019): 8563–8575, https://doi.org/10.1021/acs.chemmater.9b03775.

31. D. Pariari, S. Mehta, S. Mandal, et al., "Realizing the Lowest Bandgap and Exciton Binding Energy in a Two-Dimensional Lead Halide System," *Journal of the American Chemical Society* 145 (2023): 15896–15905, https://doi.org/10.1021/jacs.3c03300.
32. J. An, G. Chen, X. Zhu, et al., "Ambipolar Photoresponse of $CsPbX_3$–ZnO (X = Cl, Br, and I) Heterojunctions," *ACS Applied Electronic Materials* 4 (2022): 1525–1532, https://doi.org/10.1021/acsaelm.1c01085.
33. M. H. Tran, T. Park, J. Hur, "Wide-Bandgap $CaSnO_3$ Perovskite As an Efficient and Selective Deep-UV Absorber for Self-Powered and High-Performance p-i-n Photodetector," *ACS Applied Materials & Interfaces* 13 (2021): 13372–13382, https://doi.org/10.1021/acsami.0c23032.
34. G. I. Choi, C. W. Bark, and H. W. Choi, "Study on a Mixed-Cation Halide Perovskite-Based Deep-Ultraviolet Photodetector," *Coatings* 13 (2023): 248, https://doi.org/10.3390/coatings13020248.
35. T. M. H. Nguyen, S. K. Lee, S. Kim, et al., "Practical Demonstration of Deep-Ultraviolet Detection with Wearable and Self-Powered Halide Perovskite-Based Photodetector," *ACS Applied Materials & Interfaces* 13 (2021): 57609–57618, https://doi.org/10.1021/acsami.1c18099.
36. S. G. Shin, C. W. Bark, and H. W. Choi, "Study on Performance Improvements in Perovskite-Based Ultraviolet Sensors Prepared Using Toluene Antisolvent and CH_3NH_3Cl," *Nanomaterials* 11 (2021): 1000, https://doi.org/10.3390/nano11041000.
37. Y. Wang, B. Su, G. Lin, et al., "Exploring the Ruddlesden–Popper Layered Organic–Inorganic Hybrid Semiconducting Perovskite for Visible-Blind Ultraviolet Photodetection," *CrystEngComm* 24 (2022): 2258–2263, https://doi.org/10.1039/D1CE01431E.
38. C. Y. Wu, Y. X. Le, L. Y. Liang, et al., "Non-Ultrawide Bandgap $CsPbBr_3$ Nanosheet for Sensitive Deep Ultraviolet Photodetection," *Journal of Materials Science & Technology* 159 (2023): 251–257, https://doi.org/10.1016/j.jmst.2023.03.032.
39. C. H. Kang, I. Dursun, G. Liu, et al., "High-Speed Colour-Converting Photodetector with All-Inorganic CsPbBr3 Perovskite Nanocrystals for Ultraviolet Light Communication," *Light: Science & Applications* 8 (2019): 94, https://doi.org/10.1038/s41377-019-0204-4.
40. T. Zou, X. Liu, R. Qiu, et al., "Enhanced UV-C Detection of Perovskite Photodetector Arrays via Inorganic CsPbBr3 Quantum Dot Down-Conversion Layer," *Advanced Optical Materials* 7 (2019): 1801812, https://doi.org/10.1002/adom.201801812.

41. H. Li, Z. Wang, Q. Guan, et al., "Polar Three-Dimensional Organic–Inorganic Hybrid Perovskite Realize Highly Sensitive Self-Driven Ultraviolet Photodetection," *Angewandte Chemie International Edition* 64 (2025): e202500765, https://doi.org/10.1002/anie.202500765.
42. A. Ajayakumar, J. H. Kim, N. Ninkovic, et al., "Two-Dimensional Perovskite Single-Nanowire Photodetectors," *ACS Photonics* 13 (2026): 2704–2711, https://doi.org/10.1021/acsphotonics.6c00352.
